# A semisynthesis of 3'-*O*-ethyl-5,6-dihydrospinosyn J based on the spinosyn A aglycone

**DOI:** 10.3762/bjoc.13.257

**Published:** 2017-12-06

**Authors:** Kai Zhang, Shenglan Liu, Anjun Liu, Hongxin Chai, Jiarong Li, Lamusi A

**Affiliations:** 1 School of Chemistry and Chemical Engineering, Beijing Institute of Technology, 5 South Zhongguancun Street, Haidian District, Beijing, China; 2 Institute of Grassland Research of CAAS, No. 120 Wulanchabu East Street, Saihan District, Hohhot, China

**Keywords:** 3-*O*-ethyl-2,4-di-*O*-methylrhamnose, protecting groups, semisynthesis, spinetoram, spinosyn A

## Abstract

Spinetoram, a mixture of 3'-*O*-ethyl-5,6-dihydrospinosyn J (XDE-175-J, major component) and 3'-*O*-ethylspinosyn L (XDE-175-L, minor component), is a novel kind of green and efficient insecticide with a broad range of action against various insects. Nowadays, spinetoram is widely used in agriculture and food storage. This work reports a 7-step semisynthesis of 3'-*O*-ethyl-5,6-dihydrospinosyn J from spinosyn A aglycone. The C9–OH and C17–OH of the aglycone are successively connected to 3-*O*-ethyl-2,4-di-*O*-methylrhamnose and D-forosamine after selective protection and deprotection steps. Then, with 10% Pd/C as catalyst, the 5,6-double bond of the macrolide was selectively reduced to afford 3'-*O*-ethyl-5,6-dihydrospinosyn J. In addition, the 3-*O*-ethyl-2,4-di-*O*-methylrhamnose is synthesized from rhamnose which is available commercially, while the D-forosamine and aglycone are obtained via the hydrolysis of spinosyn A. High yields were obtained in each step, and all intermediates in the synthesis were characterized by ^1^H NMR, ^13^C NMR and MS techniques. This study can be helpful for developing an efficient chemical synthesis of spinetoram, and it also offers opportunities to synthesize spinosyn analogues and rhamnose derivatives.

## Introduction

**Figure 1:**
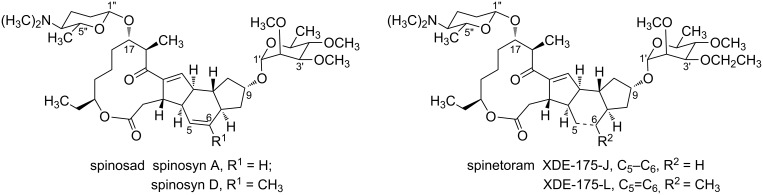
The structures of spinosad and spinetoram.

Spinosyns, a large family of secondary metabolites produced by aerobic fermentation of *Saccharopolyspora spinosa*, are a new kind of green and efficient insecticides [1]. Since the discovery of spinosyns in 1982, more than 20 species of spinosyns (named spinosyn A, B, C, D, E, F, J, K, L, M, N, O, P, Q, R, S, T, U, V, W, Y) have been found [2–5]. There are two generations of spinosyns: spinosad and spinetoram (Figure 1). Structurally, spinosad and spinetoram both consist of a unique tetracyclic nucleus which is attached to rhamnose analogues and D-forosamine [6].

Spinosad was introduced by Dow AgroSciences in 1997 for the control of insect pests in cotton under the trade name of Tracer [7]. Spinosad is a mixture of approximately 85% spinosyn A and 15% spinosyn D [8], and it can effectively control several pests including *Lepidoptera*, *Diptera*, *Thysanoptera*, *Coleoptera* and *Orthoptera* insects [8–11]. Importantly, it shows a great selectivity toward the target insects, reduced environmental persistence and low mammalian and avian toxicity [12]. Therefore, spinosad was awarded the Presidential Green Chemistry Challenge Award in 1999. However, spinosad was not effective in killing certain key pests of fruit trees and nut trees. To solve this problem, Dow AgroSciences used an "artificial neural network (ANN)" to identify analogous molecules that might be effective to control fruit-tree pests, and then, spinetoram was discovered [13].

Although the structure of spinetoram is similar to that of spinosad, spinetoram has a broader spectrum of insecticide activity and more efficient performance in contrast with spinosad [14]. Spinetoram has been widely applied in insect control and sterilization. Similar to spinosad, spinetoram can be rapidly degraded into natural components by several ways, such as photodegradation and biodegradation [15], suggesting that spinetoram is safe for the environment. In addition, spinetoram shows excellent selectivity for the target insects, while being less harmful to beneficial predators, mammals and human beings [16]. Therefore, spinetoram was awarded the Presidential Green Chemistry Challenge Award in 2008.

**Scheme 1:**
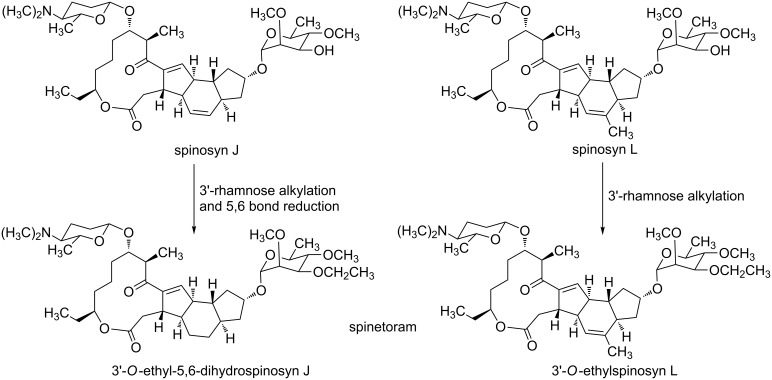
Chemical modifications of spinosyn J and spinosyn L.

**Scheme 2:**
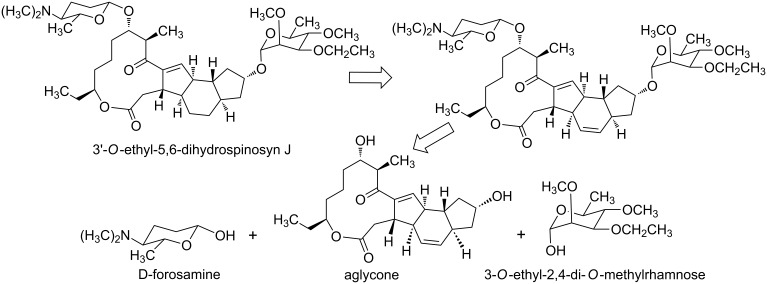
Retrosynthetic analysis of 3'-*O*-ethyl-5,6-dihydrospinosyn J.

At present, spinetoram is obtained from spinosyn J and spinosyn L by a chemical modification [13] (Scheme 1). However, the fermentation productivity of spinosyn J and spinosyn L is low, resulting in high cost. So we envisage that spinetoram can be synthesized via a chemical method. So far, several total syntheses of spinosyn A have been reported [17–22], but there are few studies on the chemical synthesis of 3'-*O*-ethyl-5,6-dihydrospinosyn J. Considering the high fermentation productivity and low cost of spinosyn A, we designed a semi-synthesis of 3'-*O*-ethyl-5,6-dihydrospinosyn J from the spinosyn A aglycone via sequential glycosylation after suitable protection and deprotection. In this synthesis route, there are three important parts: D-forosamine, 3-*O*-ethyl-2,4-di-*O*-methylrhamnose and aglycone (Scheme 2).

Since the discovery of spinosyns, many researchers have engaged in modifying the structures of spinosyns to acquire spinosyn analogues which may have more efficient insecticidal activity [23–26]. This study offers a novel approach to synthesize spinosyn analogues.

## Results and Discussion

### Hydrolysis of spinosyn A and famation of aglycone and D-forosamine

**Scheme 3:**
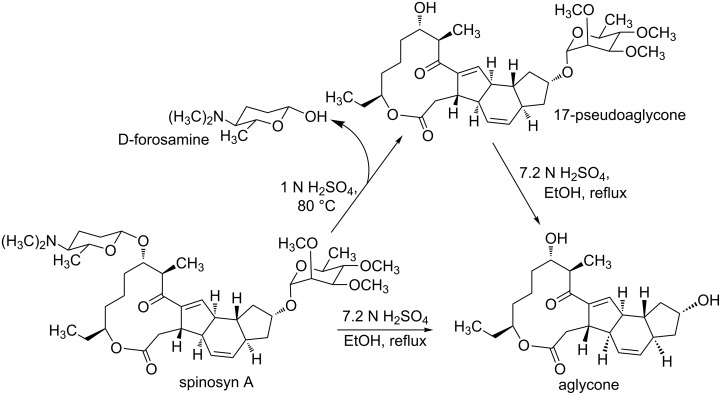
Hydrolysis of spinosyn A and formation of the aglycone and *D*-forosamine.

As reported [27], both aglycone and D-forosamine are the hydrolysis products of spinosyn A (Scheme 3). The spinosyn A aglycone and D-forosamine were prepared by a two-step hydrolysis under different acidic conditions. In addition, spinosyn A can be hydrolyzed under strong acidic conditions to give the aglycone in one step, but the separation and purification of the aglycone and D-forosamine are complicated. Therefore, the aglycone and D-forosamine are obtained through a two-step hydrolysis process in our work.

### Preparation of 3-*O*-ethyl-2,4-di-*O*-methylrhamnose

**Scheme 4:**
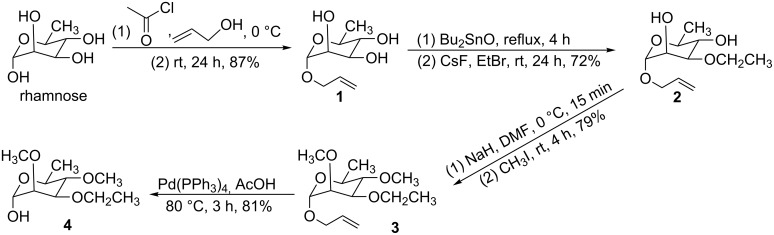
Synthesis of 3-*O*-ethyl-2,4-di-*O*-methylrhamnose (**4**).

3-*O*-Ethyl-2,4-di-*O*-methylrhamnose was synthesized from commercial rhamnose via a multistep process (Scheme 4). Rhamnose contains several hydroxy groups with similar chemical activities, so regioselective protection and alkylation are challenges. Common protecting groups of the hydroxy group at 1-position of rhamnose include allyl [28], methoxyphenyl [29] and 1-thiorhamnoside [30]. Compared with the reaction conditions of the other two protecting groups, the conditions of selective allyl protection are mild, and no irritant gas is produced during the reaction, so selective allyl protection was chosen to afford **1**. A survey of literature [31–33] shows that the combination of Bu_2_SnO and EtBr can selectively ethylate the hydroxy group at 3-position of rhamnose. The structure of **2** was also ascertained by NMR. Then compound **2** was methylated at 0 °C in the presence of iodomethane and sodium hydride to afford **3**. Finally, 3-*O*-ethyl-2,4-di-*O*-methylrhamnose (**4**) was obtained by the deprotection of **3** with Pd(PPh_3_)_4_ as catalyst in acetic acid under argon gas.

The synthetic route of 3-ethoxy-2,4-di-*O*-methylrhamnose is simple and innovative. All the reagents used in the research are available commercially, so it is likely that 3-ethoxy-2,4-di-*O*-methylrhamnose can be produced on a large scale. In addition, this method can also be used to synthesize the derivatives of rhamnose.

### Semisynthesis of 3'-*O*-ethyl-5,6-dihydrospinosyn J

**Scheme 5:**
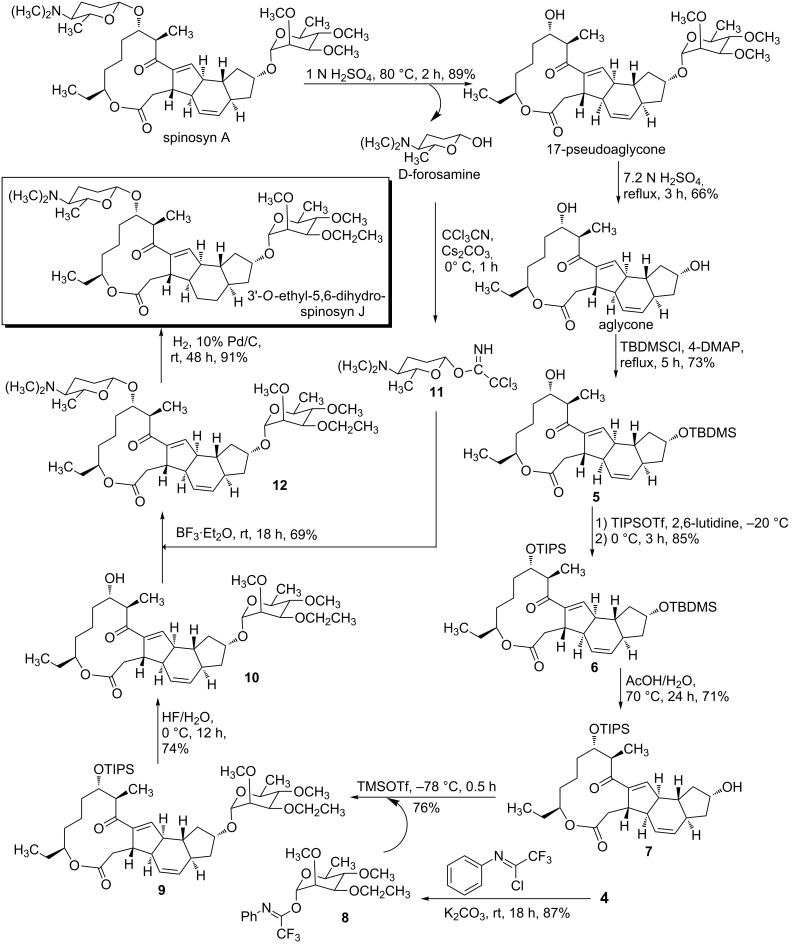
The semi-synthesis of 3'-*O*-ethyl-5,6-dihydrospinosyn J.

The C9–OH and C17–OH groups of the aglycone have similar activity, so different protection strategies are required for the hydroxy groups (Scheme 5). According to Martynow’s work [34], C9–OH can be selectively protected by TBDMSCl in the presence of 4-DMAP in dry dichloromethane, presumably due to the steric hindrance around C17. Then C17–O-protected compound **6** was prepared by using TIPSOTf as protecting reagent in the presence of 2,6-lutidine in dry dichloromethane. Due to the different stabilities of the C9–OH and C17–OH of the aglycone, the protecting groups of two hydroxy groups can be successively removed under different conditions.

Compound **6** was hydrolyzed in the presence of HOAc to afford **7**, and then the subsequent glycosidation of compound **7** with glycosyl donor **8** yielded compound **9**. Compound **8** was synthesized from **4** and 2,2,2-trifluoro-*N*-phenylethanimidoyl chloride in the presence of potassium carbonate. Compound **9** was desilylated with hydrofluoric acid at 0 °C to offer **10**, and then compound **10** was glycosylated with donor **11** to provide **12**. Compound **11** was prepared from D-forosamine and trichloroacetonitrile with Cs_2_CO_3_ as catalyst. Finally, 3'-*O*-ethyl-5,6-dihydrospinosyn J was obtained through the selective hydrogenation of **12** catalyzed by 10% Pd/C. During the final reduction step, as ascertained by NMR and mass spectrometry, only the 5,6-double bond was reduced, and the other unsaturated bonds were not affected. There is a conjugation between the 13,14-double bond and the carbonyl group, resulting in greater stability of the 13,14-double bond compared to the 5,6-double bond. Thus, the 5,6-double bond can be selectively reduced.

## Conclusion

In summary, we have developed a 7-step semisynthesis of 3'-*O*-ethyl-5,6-dihydrospinosyn J based on spinosyn A with high yields in each step. With this synthetic route, we achieved a chemoselective hydrogenation of the 5,6-double bond under mild conditions, which is of great significance for future studies on the chemical modification of spinosyns. Furthermore, this synthesis provides a chemical method to synthesize spinosyn analogues containing different substituents at C9-position and C17-position of the macrolide. Also, we developed an efficient synthesis of 3-*O*-ethyl-2,4-di-*O*-methylrhamnose from rhamnose in 4 steps, and this method can also be used to synthesize other rhamnose analogues.

## Supporting Information

File 1 Experimental and analytical data.
