# Supplementary material for: A semisynthesis of 3'-O-ethyl-5,6-dihydrospinosyn J based on the spinosyn A aglycone
Source: Beilstein J Org Chem. 2017 Dec 6;13:2603–9. doi: 10.3762/bjoc.13.257 (PMC5727846; doi:10.3762/bjoc.13.257)
Supplement: File 1 — Experimental and analytical data. [file Beilstein_J_Org_Chem-13-2603-s001.pdf]

Supporting Information

for

A semisynthesis of 3'-*O*-ethyl-5,6-dihydrospinosyn J based on the spinosyn A aglycone

Kai Zhang[1], Shenglan Liu[1], Anjun Liu[1], Hongxin Chai[1], Jiarong Li*[1] and Lamusi A*[2]

Address: [1]School of Chemistry and Chemical Engineering, Beijing Institute of Technology, 5 South Zhongguancun Street, Haidian District, Beijing, China and [2]Institute of Grassland Research of CAAS, No. 120 Wulanchabu East Street, Saihan District, Hohhot, China

Email: Jiarong Li - jrli@bit.edu.cn; Lamusi A - alms721@163.com

*Corresponding author

Experimental and analytical data

Experimental

Synthesis of 1-allylrhamnose (1)

Acetyl chloride (0.96 g, 12.23 mmol) was slowly added to allyl alcohol (0.76 g, 13.12 mmol) at 0 °C, and warmed to room temperature. After 1 h, *L*-rhamnose (0.98 g, 5.97 mmol) was added to the solution. After about 24 h, $NaHCO_3$ was added to the solution to make the pH slightly alkaline, then the mixture was extracted with ethyl

acetate thrice. The combined organic layers were dried over Na_2SO_4 and evaporated under reduced pressure. The residue was purified by column chromatography on silica gel (200–300 mesh) to afford pure **1**, yield 87%. TLC (ethyl acetate/ petroleum ether = 1:1,V:V); ^{1}H NMR (400 MHz, $CDCl_3$) *δ*: 1.28 (d, *J* = 6.0 Hz, 3H, C_5-CH_3), 3.46 (m, 1H, C_4-H), 3.64 (m, 1H, C_3-H), 3.75 (m, 1H, C_5-H), 3.95 (m, 2H, C_1-O-CH_2-), 3.97 (m, 1H, C_2-H), 4.29 (s, 3H, 3×OH), 4.77 (d, *J* = 6.0 Hz, 1H, C_1-H), 5.16 and 5.30 (m, 2H, -CH=CH_2), 5.88 (m, 1H, -CH=CH_2); ^{13}C NMR (100 MHz, $CDCl_3$) *δ*: 17.6, 88.0, 88.3, 71.0, 71.7, 72.7, 99.0, 117.5, 133.7; MS (ESI) *m/z* (%): 203.1 (M^-, 100).

Synthesis of allyl-3-*O*-ethylrhamnose (2)

Compound **1** (1.76 g, 8.63 mmol) and dibutyltin oxide (2.36 g, 9.48 mmol) were successively added to 50 ml toluene, and heated to reflux with stirring for about 4 h. The solvent was distilled off under reduced pressure, and the mixture was dried in vacuum for 1 h. Then DMF (20 ml), CsF (2.61 g, 17.18 mmol) and bromoethane (1.86 g, 17.07 mmol) were added to the mixture under argon gas. After stirring at room temperature for 24 h, the solvent was distilled off under reduced pressure, and the residue was dissolved in dichloromethane. The precipitate was filtered off, and the filtrate was washed with saturated brine and 5% aqueous $NaHCO_3$ solution. Then the combined organic layers were dried over Na_2SO_4 and evaporated under reduced pressure. The residue was purified by column chromatography on silica gel (200–300 mesh) to afford pure **2**, yield 72％. TLC(ethyl acetate/petroleum ether 1:1, V:V); ^{1}H NMR (400 MHz, $CDCl_3$) *δ*: 1.08 (m, 3H, C_5-CH_3), 1.18 (m, 3H, C_3-OC-CH_3),

3.46 (m, 2H, C_3-O-CH_2-), 3.64 (m, 1H, C_3-CH), 3.75 (m, 1H, C_5-H), 3.93 (m, 1H, C_2-CH), 3.96 (m, 1H, C_4-H), 4.14 (m, 2H, C_1-O-CH_2-), 4.29 (m, 2H, 2×OH), 4.77 (s, 1H, C1-H), 5.09 and 5.18 (2m, 2H, CH=CH_2), 5.78 (m, 1H, CH=CH_2) ; ^{13}C NMR (100 MHz, $CDCl_3$) δ: 15.6, 17.8, 65.0, 67.8, 67.9, 68.0, 71.5, 79.8, 98.6, 117.6, 133.9; MS (ESI) *m/z* (%): 255.1 (M+Na, 100).

Synthesis of allyl 3-*O*-ethyl-2,4-di-*O*-methylrhamnoside (3)

Compound **2** (0.62 g, 2.67 mmol) was dissolved in 10 ml DMF, then NaH (0.10 g, 3.21 mmol) was slowly added at 0 °C. After 15 min, CH_3I (1.01 g, 6.41 mmol) was added to the solution at room temperature. After 4 h of stirring, 5 ml ammonium hydroxide solution was added, and the mixture was extracted with ethyl acetate thrice. The combined organic layers were dried over Na_2SO_4 and evaporated under reduced pressure. The residue was purified by column chromatography on silica gel (200–300 mesh) to afford pure **3**, yield 79%. TLC (ethyl acetate/petroleum ether 1:10, V:V); ^{1}H NMR (400 MHz, $CDCl_3$) δ: 1.18 (m, 6H, C_5-CH_3, C_3-OC-CH_3), 3.10 (m, 1H, C_4-H), 3.39 (m, 9H, C_2-OCH_3, C_3-OCH_3, C_3-OCH_2-, C_3-H), 3.71 (m, 2H, C_1-OCH_2-), 3.96 (m, 1H, C_2-H), 4.16 (m, 1H, C_5-H), 4.84 (s, 1H, C_1-CH), 5.20 and 5.30 (2m, 2H, CH=CH_2), 5.93 (m, 1H, CH=CH_2) ; ^{13}C NMR (100 MHz, $CDCl_3$) δ: 15.7, 17.8, 59.2, 61.0, 65.6, 67.8, 67.9, 78.2, 79.7, 82.0, 96.2, 117.3, 133.9; MS (ESI) *m/z* (%): 283.2 (M+Na, 100).

Synthesis of 3-*O*-ethyl-2,4-di-*O*-methylrhamnose (4)

Compound **3** (0.27 g, 1.04 mmol) and $Pd(PPh_3)_4$ (0.58 g, 0.51 mmol) were added to 5ml acetic acid under argon gas, and heated to 80 °C with stirring for 3 h. When the

reaction was complete (by TLC monitoring), the solvent was distilled off under reduced pressure, and the residue was purified by column chromatography on silica gel (200–300 mesh) to afford pure **4**, yield 81%. TLC (ethyl acetate/petroleum ether 1:3, V:V); 1H NMR (400 MHz, $CDCl_3$) *δ*: 1.12-1.20 (m, 6H, C_5-CH_3, C_3-OC-CH_3), 3.40 (s, 3H, C_4-OCH_3), 3.62 (s, 3H, C_2-OCH_3), 3.92 (m, 2H, C2-H, C4-H), 4.07 (m, 2H, C_3-OCH_2-), 4.74 (s, 1H, OH), 5.07-5.20 (m, 2H, C3-H, C5-H), 5.80 (m, 1H, C_1-H); 13C NMR (100 MHz, $CDCl_3$) *δ*: 133.89, 117.62, 98.59, 79.77, 71.54, 68.04, 67.89, 64.99, 17.77, 15.62; MS (ESI) m/z (%): 243.2 (M+Na, 100).

Synthesis of C9-OTBDMS-substituted aglycone 5

The aglycone (3.11 g, 7.71 mmol） was added to 60 ml dry CH_2Cl_2, then 4-dimethylaminopyridine (4-DMAP, 1.83 g, 14.98 mmol) and *tert*-butyldimethylsilyl chloride (TBDMSCl, 1.39 g, 9.22 mmol) were successively added. After addition of all the reagents, the mixture was heated to reflux with stirring for 5 h. When the reaction was completed (by TLC monitoring), the solution was diluted with CH_2Cl_2 and washed with saturated sodium bicarbonate solution thrice. The combined organic layers were dried over Na_2SO_4 and evaporated under reduced pressure. The residue was purified by column chromatography on silica gel (200–300 mesh) to afford pure **5**, yield 73%. TLC (ethyl acetate/petroleum ether 1:5, V:V); ^{1}H NMR(400 MHz, $CDCl_3$) *δ*: 0.03 (s, 6H, $Si(CH_3)_2$), 0.81 (t, 3H, C_{23}-H), 0.87 (s, 9H, 3×CH_3), 1.20 (d, 3H, C_{24}-H), 1.25 (m, 1H, C_{11}-H), 1.38-1.79(m, 12H, C_8-H C_{10}-H, C_{18}-H, C_{19}-H, C_{20}-H, C_{22}-H), 2.22 (H, m, C_7-H). 2.39 (d, 1H, *J* = 13.4Hz, C_2-H), 2.87 (m, 1H, C_{12}-H), 2.99 (m, 1H, C_{16}-H), 3.12 (d, 1H, *J*=13.4Hz, C_2-H), 3.19 (m, 1H, C_3-H), 3.43 (m, 1H,

C_4-H), 3.67 (m, 1H, C_{17}-H), 4.34 (m, 1H, C_9-H), 4.69 (s, 1H, C_{21}-H), 5.77(m, 1H, C_5-H), 5.85(m, 1H, C_6-H), 6.79 (s, 1H, C_{13}-H); ^{13}C NMR (100MHz, $CDCl_3$) *δ*: 4.51, 9.16, 15.9, 21.8, 26.1, 28.6, 30.2, 33.0, 34.2, 35.0, 40.8, 40.8, 41.4, 41.7, 46.4, 47.8, 48.2, 49.7, 72.7, 72.8, 77.1, 128.5, 130.1, 144.3, 148.2, 172.8, 202.7; MS (ESI) cal for $C_{30}H_{48}O_5Si$ $[M+Na]^+$ 517.33428, found $[M+Na]^+$ 517.33417.

Synthesis of C9-OTBDMS- and C17-OTIPS-substituted aglycone 6

Compound **5** (0.99 g, 1.92 mmol) was dissolved in 60 ml CH_2Cl_2, then 2,6-lutidine(0.42 g, 4.01 mmol) and triisopropylsilyl trifluoromethanesulfonate (TIPSOTf, 0.66 g, 2.16 mmol) were successively added at –20 °C. After addition of all the reagents, the mixture was kept at 0 °C and stirred for about 3 h. Then the mixture was diluted with CH_2Cl_2 and washed with saturated sodium bicarbonate solution thrice. The combined organic layers were dried over Na_2SO_4 and evaporated under reduced pressure. The residue was purified by column chromatography on silica gel (200–300 mesh) to afford pure **6**, yield 85%. TLC (ethyl acetate/petroleum ether 1:10, V:V); ^{1}H NMR (400 MHz, $CDCl_3$) *δ*:; 1.04 (s, 18H, Si-C-CH_3), 1.25 (m, 3H, Si-CH-), 0.03 (s, 6H, $Si(CH_3)_2$), 0.81 (t, 3H, C_{23}-H), 0.87 (s, 9H, CH_3), 1.24 (m, 3H, C_{24}-H), 1.19 (m, 1H, C_{11}-H), (1.37-1.83, 2.21) (m, 2H×6, C_8-H, C_{10}-H, C_{18}-H, C_{19}-H, C_{20}-H, C_{22}-H), 2.24 (m, 1H, C_7-H). 2.42 (d, 1H, *J* = 10.1Hz, C_2-H), 2.88 (m, 1H, C_{12}-H), 3.02 (m, 1H, C_{16}-H), 3.08 (d, 1H, *J*=10.1Hz, C_2-H), 3.22 (m, 1H, C_3-H), 3.46 (m, 1H, C_4-H), 4.05 (m, 1H, C_{17}-H), 4.34 (m, 1H, C_9-H), 4.64 (s, 1H, C_{21}-H), 5.76(m, 1H, C_5-H), 5.84(m, 1H, C_6-H), 6.87(1H, s, C_{13}-H); ^{13}C NMR (100 MHz, $CDCl_3$) *δ*; -4.65, 9.47, 13.0, 18.2, 18.5, 26.0, 19.5, 28.0, 31.3, 34.9, 36.8, 40.9,

40.9 41.2 41.6, 46.9, 47.8, 48.8, 49.8, 72.8, 74.7, 76.0, 128.9, 130.0, 143.5, 148.2, 172.7, 203.6; MS (ESI) cal for $C_{39}H_{68}O_5Si_2$ $[M+H]^+$ 673.46780, found $[M+H]^+$ 673.46775.

Synthesis of C17-OTIPS-substituted aglycone 7

Compound **6** (0.71 g, 1.04 mmol) was added to a solvent mixture of 20 ml THF, 40 ml HOAc and 25 ml H_2O, and then the mixture was heated to 70 °C with stirring for about 24 h. Then THF was evaporated under reduced pressure. The mixture was diluted with H_2O, washed with saturated sodium bicarbonate solution and extracted with EtOAc thrice. The combined organic layers were dried over Na_2SO_4 and evaporated under reduced pressure. The residue was purified by column chromatography on silica gel (200–300 mesh) to afford pure **7**, yield 71%. TLC (ethyl acetate/petroleum ether 1:5, V:V); ^{1}H NMR (400 MHz, $CDCl_3$) δ: 1.00 (s, 18H, CH_3), 1.25 (t, 3H, J=8.6Hz, CH), 0.81 (t, 3H, *J=7.5Hz*, C_{23}-H), 0.94 (m, 3H, C_{24}-H), 1.23 (m, 1H, C_{11}-H), (1.42-1.88, 2.36) (m, 12H, C_8-H, C_{10}-H, C_{18}-H, C_{19}-H, C_{20}-H, C_{22}-H), 2.26 (m, 1H, C_7-H). 2.41 (d, 1H, J= 5.0Hz, one of C_2-H), 2.92 (m, 1H, C_{12}-H), 3.04 (m, 1H, C_{16}-H), 3.08 (d, 1H, *J=5.0Hz*, one of C_2-H), 3.23 (m, 1H, C_3-H), 3.50 (m, 1H, C_4-H), 4.03 (m, 1H, C_{17}-H), 4.46 (m, 1H, C_9-H), 4.65 (s, 1H, C_{21}-H), 5.81(m, 1H, C_5-H), 5.87(m, 1H, C_6-H), 6.88 (s, 1H, C_{13}-H); ^{13}C NMR (100 MHz, $CDCl_3$) δ:13.0, 18.4, 9.6, 18.6, 19.5, 28.1, 31.3, 34.8, 36.8, 40.1, 40.8, 41.2, 41.7, 47.2, 47.8, 48.9, 49.7, 72.5, 74.7, 75.9, 129.3, 129.6, 143.6, 147.8, 172.7, 203.6; MS (ESI) cal for $C_{33}H_{54}O_5Si$ $[M+H]^+$ 559.38133, found $[M+H]^+$ 559.38164.

Synthesis of (3-*O*-ethyl-2,4-di-*O*-methyl-L-rhamnopyranosyl)-2,2,2-trifluoro-*N*-phenylacetimidate (8)

Compound **4** (0.91 g, 4.09 mmol) was dissolved in 5ml acetone, and then 2,2,2-trifluoro-*N*-phenylethanimidoyl chloride (0.87 g, 4.19 mmol) and potassium carbonate (0.58 g, 4.19 mmol) were successively added. After stirring at room temperature for about 18 h, the mixture was evaporated under reduced pressure. The residue was purified by column chromatography on silica gel (200–300 mesh) to afford pure **8**, yield 87%. TLC (ethyl acetate/petroleum ether 4:1, V:V); ^{1}H NMR (400 MHz, $CDCl_3$) δ: 1.31 (m, 3H, CH_3), 1.35 (m, 3H, CH_3), 3.15 (m, 1H, CH), 3.22 (m, 1H, CH), 3.50 (s, 3H, CH_3), 3.59 (s, 3H, CH_3), 3.61 (m, 2H, CH_2), 3.69 (m, 1H, CH), 3.75 (m, 1H, CH), 5.25 (s, 1H, CH); 6.88(m, 1H, CH), 7.14 (s, 1H, CH), 7.33 (m, 1H, CH), 7.44 (s, 1H, CH); 7.59 (s, 1H, CH) ; ^{13}C NMR (100 MHz, $CDCl_3$) δ: 15.6, 17.8, 59.2, 61.1, 67.8, 70.7, 79.7, 82.0, 96.2, 117.3, 119.5, 120.4, 125.4, 129.2, 129.4,133.9, 143.6; MS (ESI) cal for $C_{18}H_{24}F_3NO_5$ $[M+Na]^+$ 414.14988, found $[M+Na]^+$ 414.15034.

Synthesis of C17-OTIPS-substituted 3'-*O*-ethyl-5,6-dihydrospinosyn J analogue 9

Compound **8** (0.16 g, 0.29 mmol) and **7** (0.12 g, 0.31 mmol) were added to 5ml CH_2Cl_2, and then one drop of TMSOTF (about 0.05 ml) was added at –78 °C. With stirring for 0.5 h, the solution turned red, and was then quenched with sodium chloride solution. The mixture was extracted with EtOAc thrice, and the combined organic layers were dried over Na_2SO_4 and evaporated under reduced pressure.

The residue was purified by column chromatography on silica gel (200–300 mesh) to afford pure **9**, yield 76%. TLC (ethyl acetate/petroleum ether 1:4, V:V); ^{1}H NMR (400 MHz, $CDCl_3$) δ: 0.83 (t, 3H, J=7.5Hz, C_{23}-H), 0.92 (m, 1H, C_{11}-H), 1.23(d, 3H, J = 6.4 Hz, C_{24}-H), (1.37-1.93, 2.27) (2H×6, C_8-H, C_{10}-H, C_{18}-H, C_{19}-H, C_{20}-H, C_{22}-H), 2.40 and 3.10 (m, 2H, C_2-H), 2.17 (m, H, C_7-H), 2.88 (m, 2H, C_{12}-H), 3.03 (s, 1H, C_{16}-OH), 3.22 (m H, C_{12}-H), 3.45 (m, H, C_4-H), 4.06 (m, 1H, C_{17}-H), 4.32 (m, 1H, C_{9}-H), 4.67 (m, 1H, C_{21}-H), 5.84(m, 1H, C_5-H), 5.86(m, 1H, C_6-H), 6.85 (s, 1H, C_{13}-H); 1.28 (d, 3H, J= 6.5 Hz, $C_{5'}$-CH_3), 1.30 (m, 2H, O-CH_2-), 3.14 (m, 1H, $C_{4'}$-H), 3.47 (m, 1H, $C_{3'}$-H), 3.50 (s, 3H, $C_{2'}$-OCH_3), 3.50 (s, 3H, $C_{5'}$-OC-CH_3), 3.73 (m, 1H, $C_{2'}$-H), 3.70 (m, 1H, $C_{5'}$-H), 3.57 (s, 3H, $C_{4'}$-OCH_3), 4.85 (s, 1H, $C_{1'}$-H), 1.09 (s, 18H, Si-C-CH_3), 1.25 (m, 3H, Si-CH-); ^{13}C NMR (100 MHz, CDCl3) δ: 9.6, 13.0, 15.9, 17.9, 18.4, 18.6, 19.5, 28.1, 31.4, 34.9, 36.5, 36.8, 37.7, 41.2, 41.6, 46.6, 47.8, 48.4, 49.6, 59.4, 61.2, 66.7, 68.1, 74.7, 76.0, 77.4, 78.4, 82.2, 82.3, 95.8, 129.3, 129.6, 143.6, 147.8, 172.7, 203.6; MS (ESI) cal for $C_{43}H_{72}O_9Si$ $[M+Na]^+$ 783.48378, found $[M+Na]^+$ 783.48396.

Synthesis of 17-pseudoaglycone of 3'-*O*-ethyl-5,6-dihydrospinosyn J analogue 10

Compound **9** (0.21 g, 0.28 mmol) was added to a solvent mixture of 15 ml acetonitrile and 2.5 ml 40% hydrofluoric acid at 0 °C. After stirring for about 12 h, the mixture was diluted with H_2O, washed with saturated sodium bicarbonate solution and extracted with EtOAc thrice. The combined organic layers were dried over Na_2SO_4 and evaporated under reduced pressure. The residue was purified by column chromatography on silica gel (200–300 mesh) to afford pure **10**, yield 74%.

TLC (ethyl acetate/petroleum ether 1:1, V:V); ^{1}H NMR (400 MHz, $CDCl_3$) δ: 0.82 (t, 3H, C_{23}-H), 0.92 (m, 1H, C_{11}-H), 1.23 (d, 3H, J = 6.4 Hz, C_{24}-H), 1.37-1.96 and 2.27 (m, 12H, C_8-H, C_{10}-H, C_{18}-H, C_{19}-H, C_{20}-H, C_{22}-H), 2.41 and 3.12 (m, 2H, C_2-H), 2.17 (m, 1H, C_7-H), 2.88 (m, 2H, C_{12}-H), 3.03 (m, 1H, C_{16}-H), 3.21 (m, 1H, C_{12}-H), 3.46 (m, 1H, C_4-H), 3.62 (m, 1H, C_{17}-H), 4.32 (m, 1H, C_9-H), 4.69 (m, 1H, C_{21}-H), 5.81 (m, 1H, C_6-H), 5.87 (m, 1H, C_5-H), 6.78 (s, 1H, C_{13}-H); 1.28 (d, 3H, J=6.5Hz, $C_{5'}$-CH_3), 1.31 (m, 2H, $C_{4'}$-O-CH_2-), 3.14 (m, 1H, $C_{4'}$-H), 3.48 (m, 1H, $C_{3'}$-H), 3.50 (s, 3H, $C_{2'}$-OCH_3), 3.50 (s, 3H, $C_{5'}$-O-C-CH_3), 3.73 (m, 1H, $C_{2'}$-H), 3.69 (m, 1H, C_5'-H), 3.57 (s, 3H, $C_{4'}$-$OCCH_3$), 4.83 (s, 1H, $C_{1'}$-H); ^{13}C NMR (100 MHz, $CDCl_3$) δ: 9.5, 15.9, 18.0, 18.6, 21.7, 28.5, 30.2, 34.2, 35.0, 36.4, 37.5, 41.3, 41.6, 461, 47.7, 48.2, 49.6, 59.4, 61.2, 66.7, 68.2, 72.8, 76.2, 77.1, 78.6, 79.8, 82.3, 95.9, 128.9, 129.5, 144.5, 147.6, 172.8, 202.9; MS (ESI) cal for $C_{34}H_{52}O_9$ $[M+Na]^+$ 627.35035, found $[M+Na]^+$ 627.35058.

Synthesis of forosamineyl trichoroacetimidate (11)

D-Forosamine (0.18 g, 1.13 mmol) was added to 10 ml CH_2Cl_2, and then trichloroacetonitrile (0.42 g, 2.91 mmol) and Cs_2CO_3 (0.11g, 0.34mmol) were successively added at 0 °C. After stirring for 1 h, the mixture was diluted with CH_2Cl_2 and washed with saturated sodium bicarbonate solution. The combined organic layers were dried over Na_2SO_4, and then evaporated under reduced pressure. The residue was used directly in the next reaction.

Synthesis of 3'-*O*-ethyl-5,6-dihydrospinosyn J analogue 12

Compound **10** (0.12 g, 0.19 mmol) was added to 20 ml CH_2Cl_2, then **11** (0.09 g, 0.30 mmol) and 0.05 ml trifluoride etherate were added at room temperature. After stirring

for 18 h, the mixture was diluted with CH_2Cl_2 and washed with saturated sodium bicarbonate solution. The combined organic layers were dried over Na_2SO_4 and evaporated under reduced pressure. The residue was purified by column chromatography on silica gel (200–300 mesh) to afford pure **12**, yield 69%. TLC (methanol/dichloromethane 1:5, V:V); ^{1}H NMR (400 MHz, $CDCl_3$) δ: 6.70(s, 1H, C_{13}-H), 5.82(m, 1H, C_6-H), 5.74(m, 1H, C_5-H), 4.78(s,1H, $C_{1'}$-H), 4.60(m, 1H, C_{21}-H), 4.35(d, J=3.8Hz, 1H, $C_{1''}$-H), 4.24(m, 1H, C_9-H), 3.56(m, 1H, $C_{2'}$-H), 3.48-3.38(m, 13H, C_{17}-H, $C_{5'}$-H, C_4-H, $C_{4'}$-OCH_3, $C_{2'}$-OCH_3, $C_{3'}$-OCH_2-, $C_{3'}$-H, $C_{5''}$-H), 3.22(m, 1H, C_{16}-H), 3.08-3.02(m, 2H, one of C_2-H, C_3-H), 2.94(m, 1H, $C_{4'}$-H), 2.80(m, 1H, C_{12}-H), 2.34(m, 1H, one of C_2-H), 2.21-2.09(m, 10H, C_{10}-H, C_7-H, $C_{4''}$-H, $N(CH_3)_2$), 1.91-1.66(m, 5H, one of C_8-H, one of $C_{2''}$-H, one of $C_{3''}$-H, one of C_8-H, one of C_{19}-H), 1.47-1.28(m, 10H, C_{18}-H, one of C_{20}-H, C_{22}-H, one of $C_{2''}$-H, one of $C_{3''}$-H, $C_{3'}$-OC-CH_3), 1.21-1.17(m, 11H, one of C_{19}-H, one of C_{20}-H, $C_{5'}$-CH_3, C_{16}-CH_3), 0.85(m, 1H, C_{11}-H), 0.75(t, J = 7.2Hz, 3H, C_{23}-H); ^{13}C NMR (101 MHz, $CDCl_3$) δ 202.76, 172.43, 147.40, 144.11, 129.26, 128.77, 103.40, 95.43, 82.22, 81.05, 80.53, 77.67, 76.61, 76.03, 73.61, 67.88, 64.84, 60.84, 60.26, 58.94, 57.63, 49.38, 47.62, 47.57, 46.00, 41.47, 41.12, 40.65, 37.34, 36.25, 34.27, 30.92, 30.06, 28.36, 21.59, 20.95, 18.90, 18.33, 17.75, 16.08, 14.15, 9.30; MS (MALDI) cal for $C_{42}H_{67}NO_{10}$ $[M+Na]^+$ 768.465718, found $[M+Na]^+$ 768.465844.

Synthesis of 3'-*O*-ethyl-5,6-dihydro spinosyn J

Compound **12** (0.09 g, 0.12 mmol) was dissolved in 20 ml methanol, and then 10% Pd/C (0.0186 g, 0.0175 mmol) was added. The mixture was stirred under hydrogen

at room temperature. After about 48 h of stirring, the mixture was filtered. The filtrate was evaporated under reduced pressure, and the residue was purified by column chromatography on silica gel (200–300 mesh) to afford pure 3'-*O*-ethyl-5,6-dihydro spinosyn J, yield 91%. TLC (methanol/dichloromethane 1:8, V:V); ^{1}H NMR (400 MHz, $CDCl_3$) δ: 6.86(s, 1H, C_{13}-H), 4.84(s,1H, $C_{1'}$-H), 4.60(m, 1H, C_{21}-H), 4.44(d, J=8.4Hz, 1H, $C_{1''}$-H), 4.21(m, 1H, C_9-H), 3.73(m, 1H, $C_{2'}$-H), 3.65(m, 1H, $C_{5''}$-H), 3.62(m, 1H, C_{17}-H), 3.57-3.48(m, 11H, $C_{5'}$-H, C_4-H, $C_{4'}$-OCH_3, $C_{2'}$-OCH_3, $C_{3'}$-OCH_2-, $C_{3'}$-H), 3.44(m, 1H, C_{16}-H), 3.42-3.40 (m, 2H, one of C_2-H, C_3-H), 3.11(m, 1H, $C_{4'}$-H), 2.81(m, 1H, C_{12}-H), 2.35(m, 1H, one of C_2-H), 2.30-2.22(m, 10H, C_{10}-H, C_7-H, $C_{4''}$-H, $N(CH_3)_2$), 1.98-1.81(m, 5H, one of C_8-H, one of $C_{2''}$-H, one of $C_{3''}$-H, one of C_8-H, one of C_{19}-H), 1.57-1.43(m, 12H, C_{18}-H, one of C_{20}-H, C_{22}-H, one of $C_{2''}$-H, one of $C_{3''}$-H, $C_{3'}$-OC-CH_3, one of C_5-H, one of C_6-H), 1.28-1.16(m, 13H, one of C_{19}-H, one of C_{20}-H, $C_{5'}$-CH_3, C_{16}-CH_3, one of C_5-H, one of C_6-H, $C_{5''}$-CH_3), 1.03(m, 1H, C_{11}-H), 0.82(t, J = 7.4Hz, 3H, C_{23}-H); ^{13}C NMR (101 MHz, $CDCl_3$) δ 203.28, 172.48, 149.45, 145.07, 103.22, 95.73, 82.11, 80.28, 79.56, 78.48, 75.72, 75.55, 73.35, 68.14, 67.83, 65.44, 64.80, 60.84, 59.09, 49.98, 47.79, 46.44, 43.15, 40.92, 40.52, 39.47, 38.68, 37.94, 34.21, 32.94, 30.76, 29.91, 28.35, 26.94, 24.43, 21.83, 18.89, 18.69, 17.73, 15.92, 15.64, 9.25. MS (MALDI) cal for $C_{42}H_{69}NO_{10}$ $[M+Na]^+$ 770.481368, found $[M+Na]^+$ 770.481264.

Analytical data

^{1}H NMR of **1**

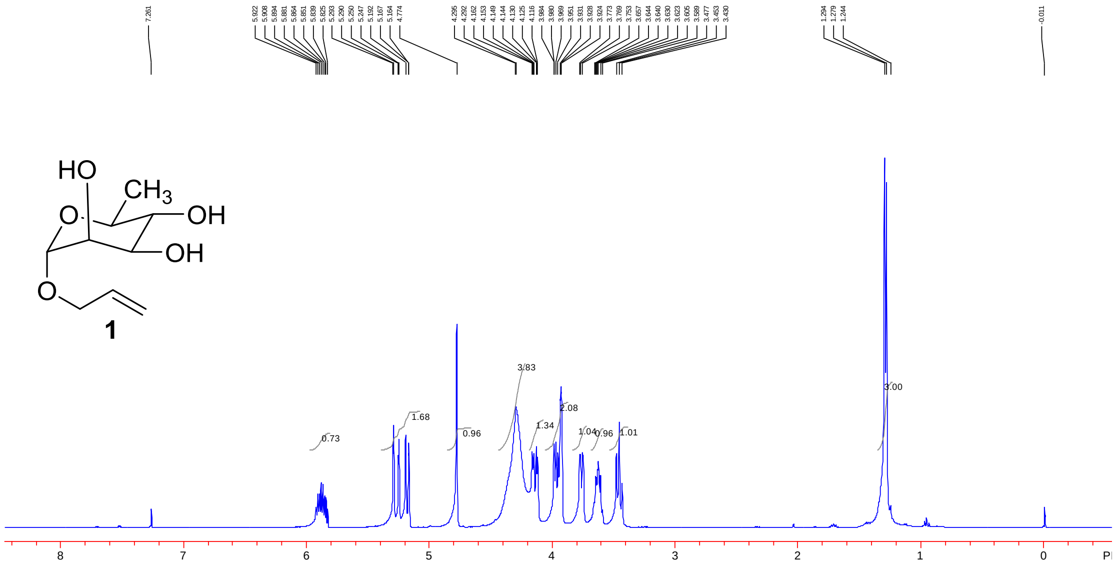

^{13}C NMR of **1**

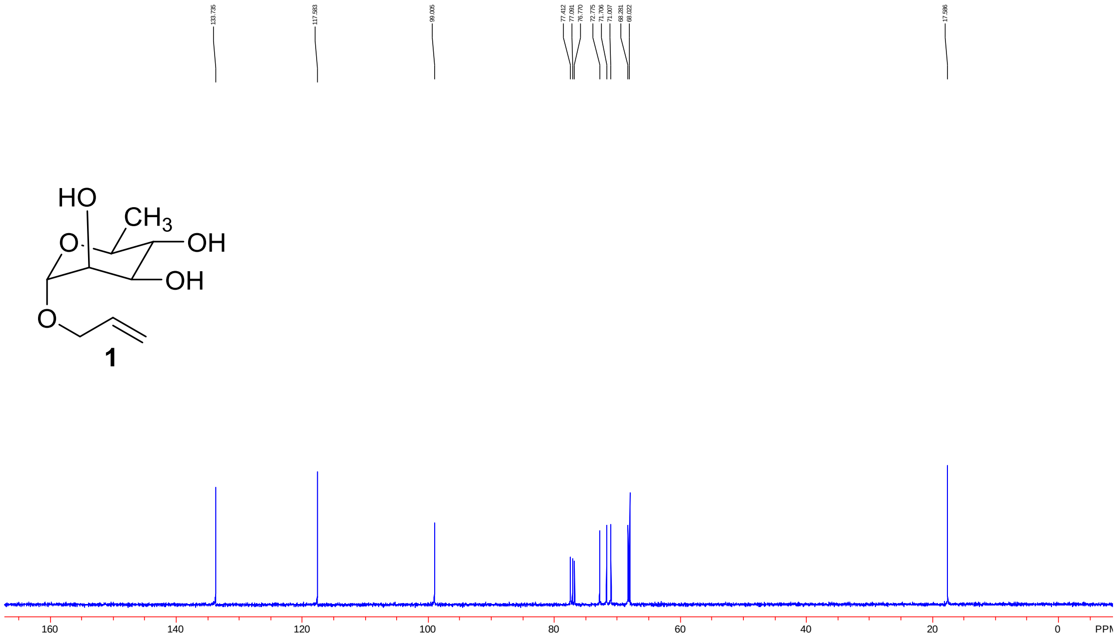

^{1}H NMR of **2**

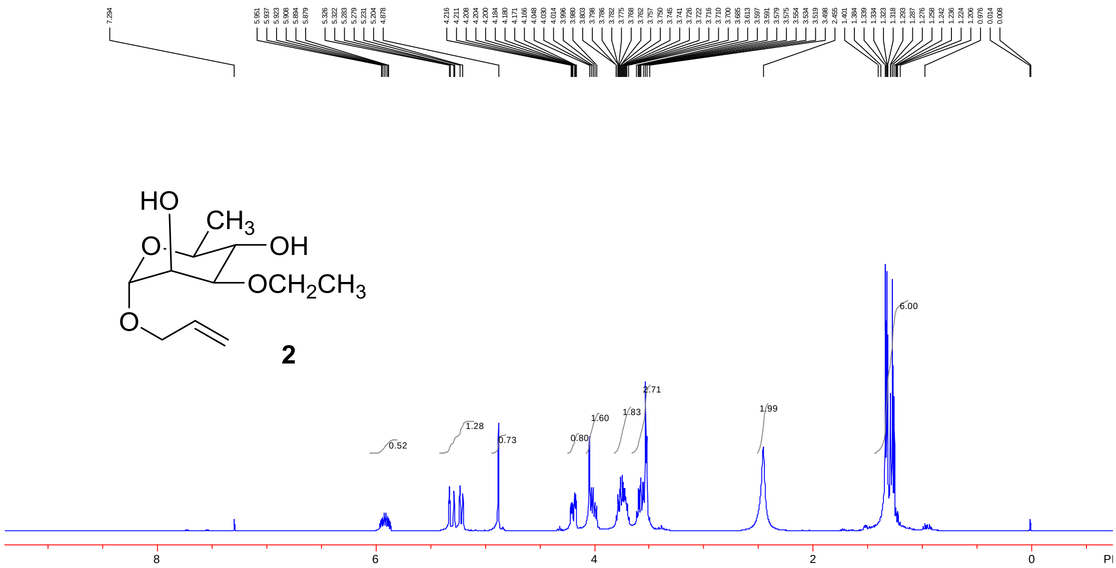

^{13}C NMR of **2**

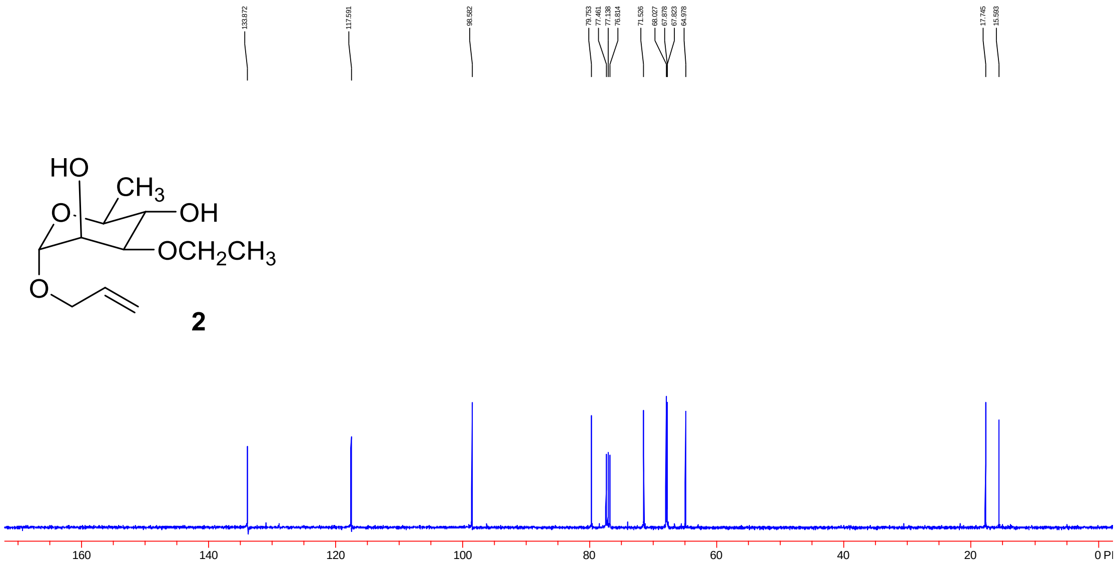

^{1}H NMR of **3**

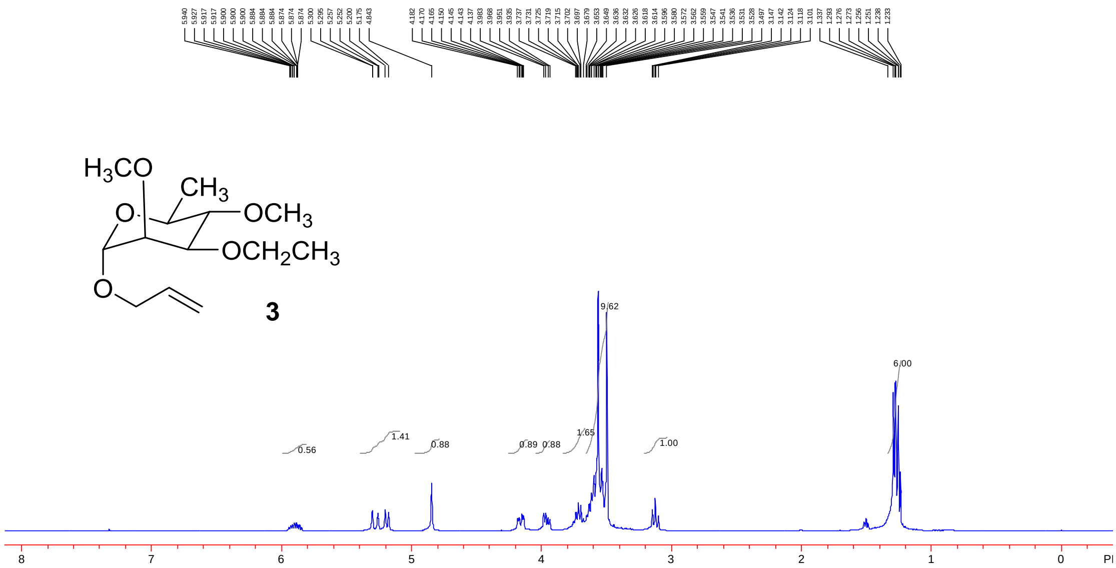

^{13}C NMR of **3**

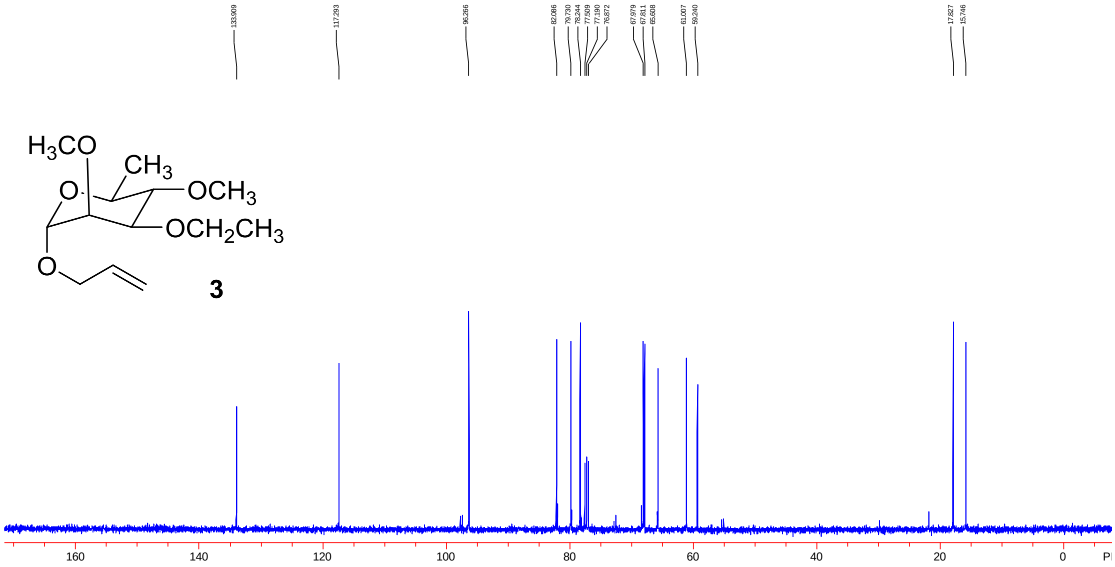

^{1}H NMR of **4**

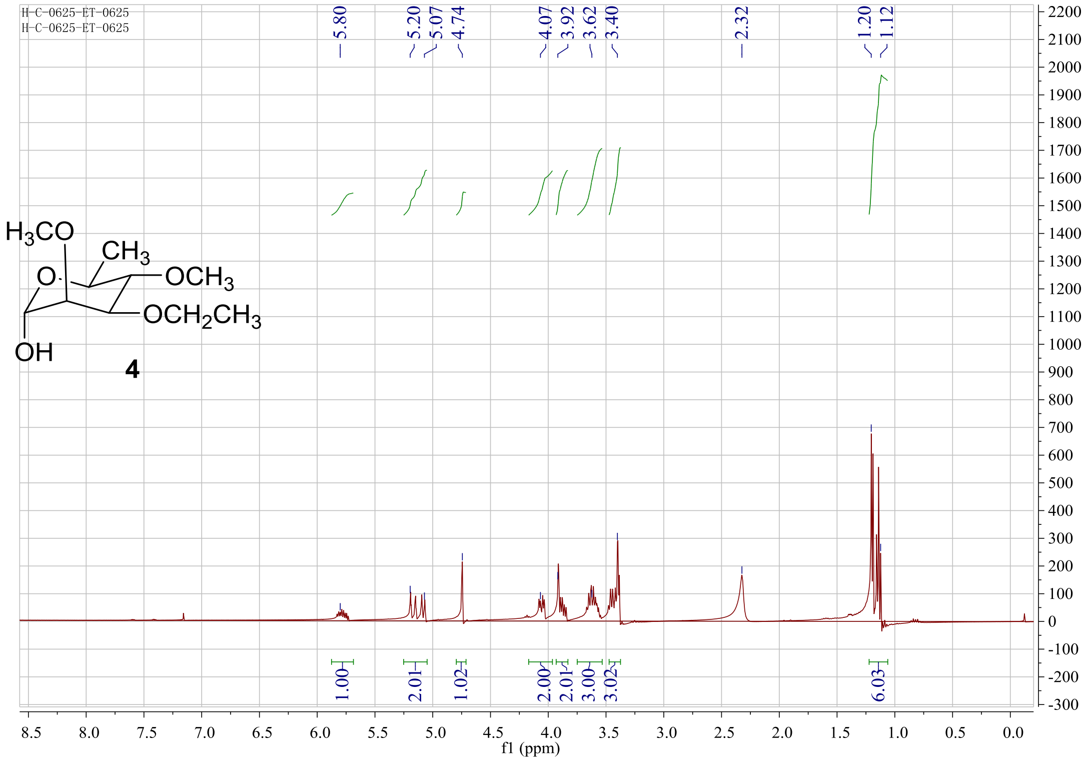

^{13}C NMR of **4**

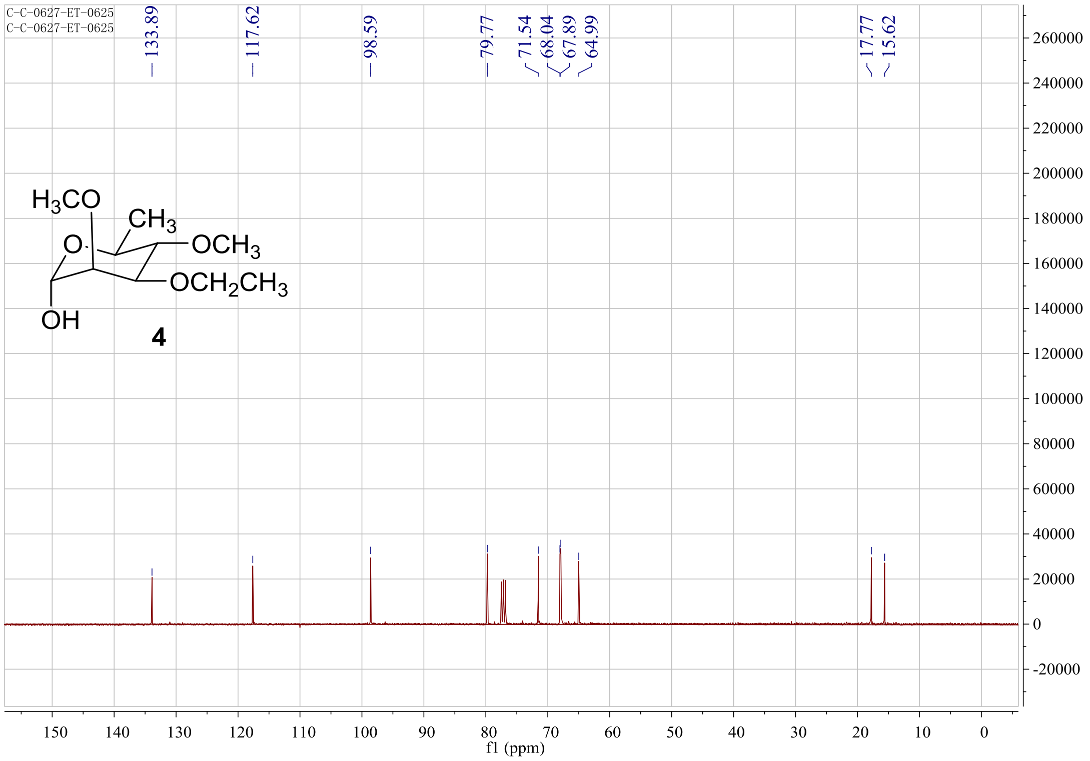

^{1}H NMR of **5**

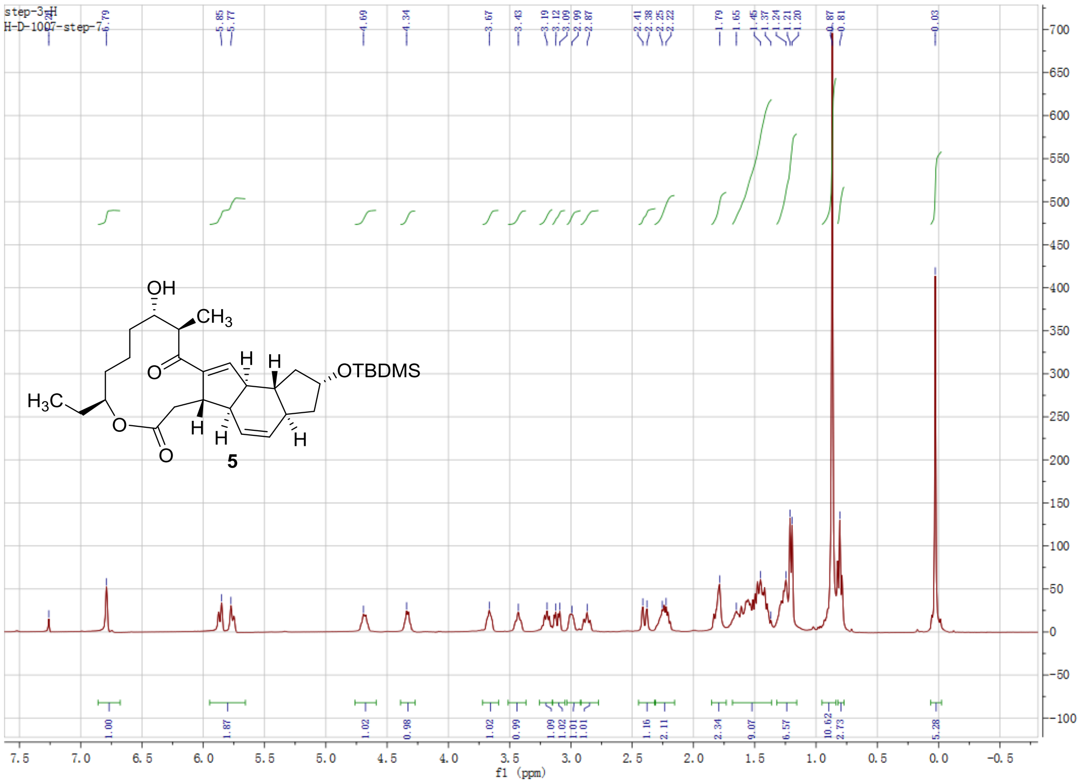

^{13}C NMR of **5**

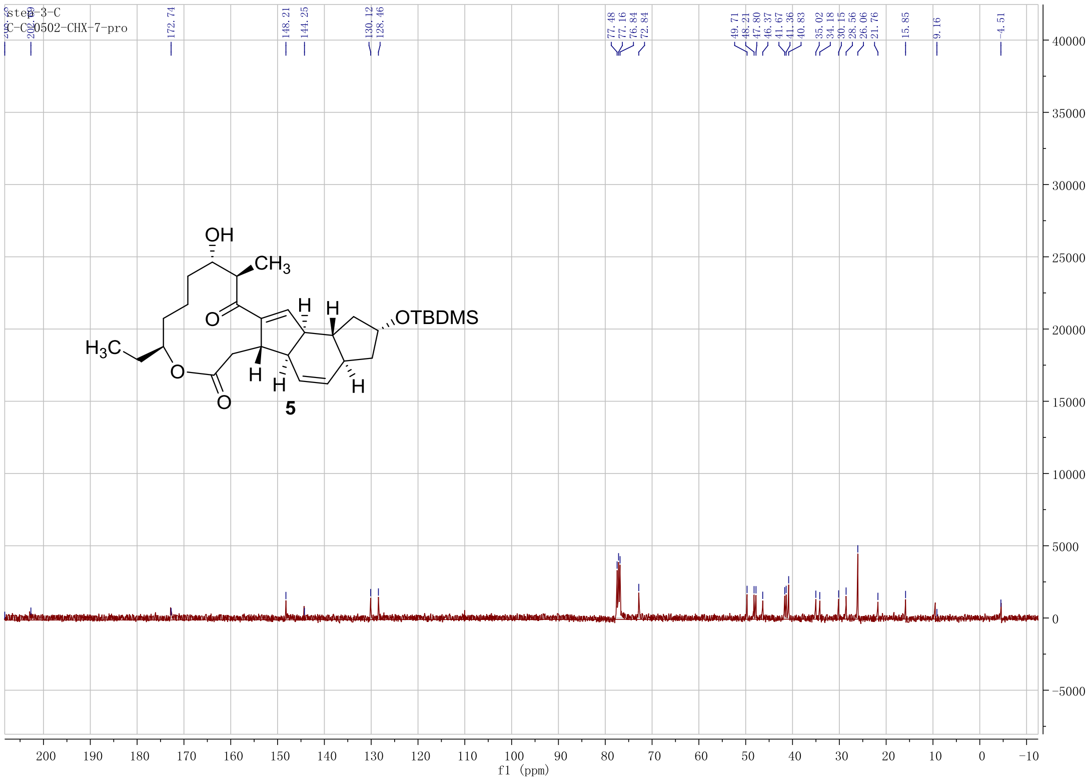

^{1}H NMR of **6**

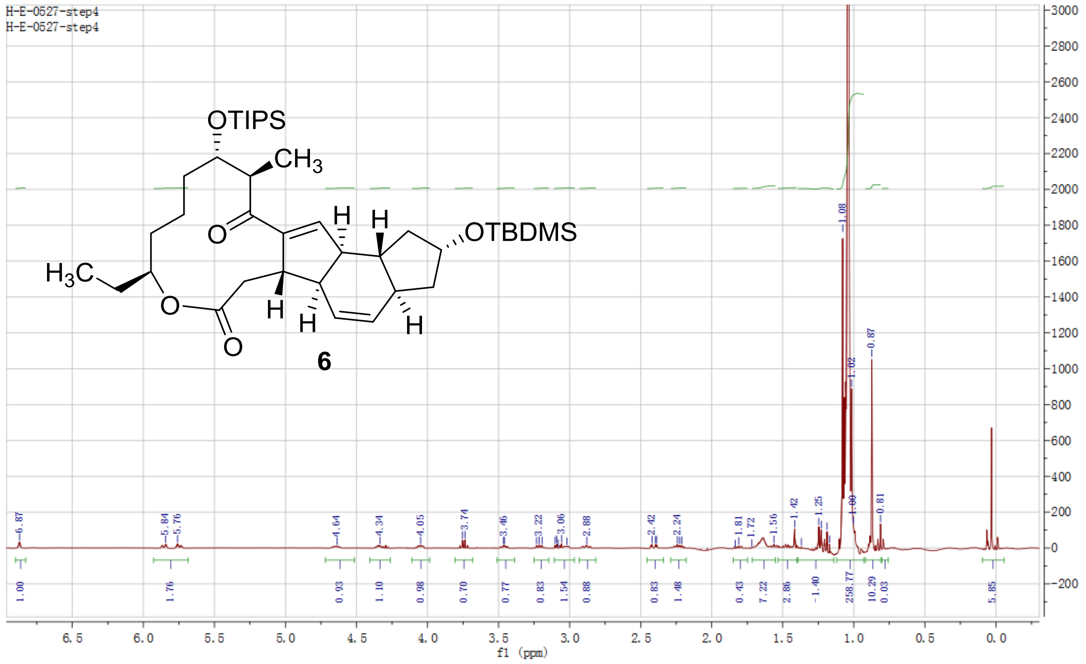

^{13}C NMR of **6**

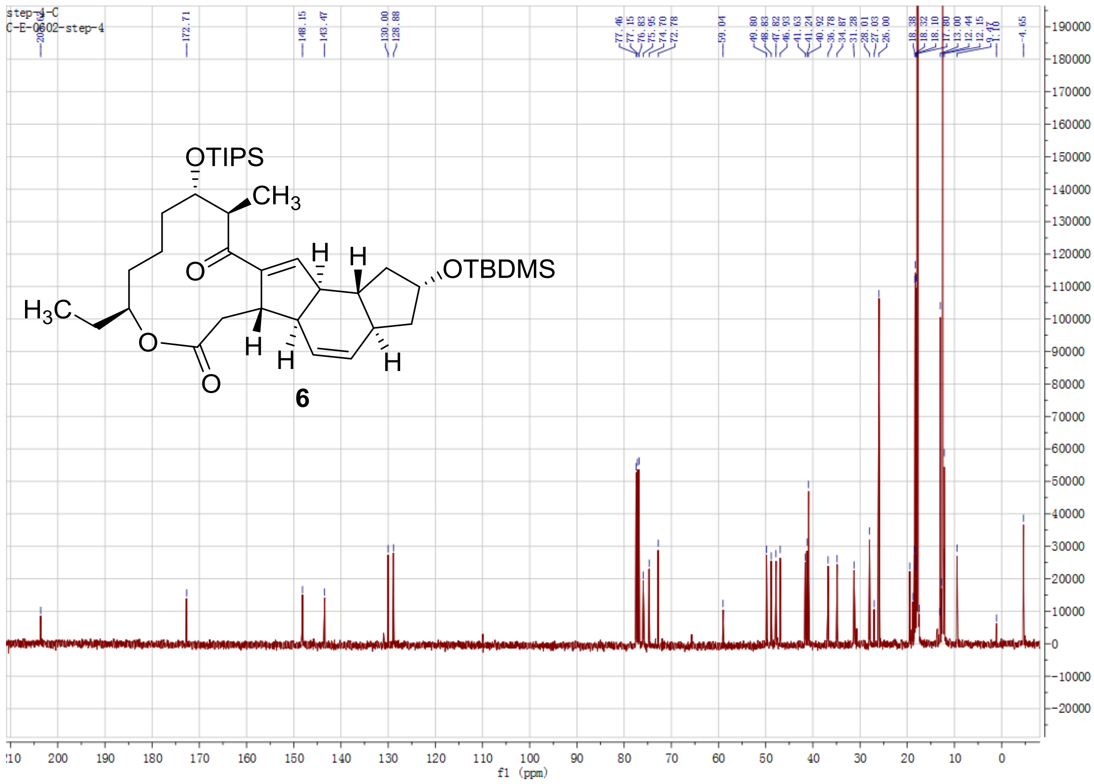

^{1}H NMR of **7**

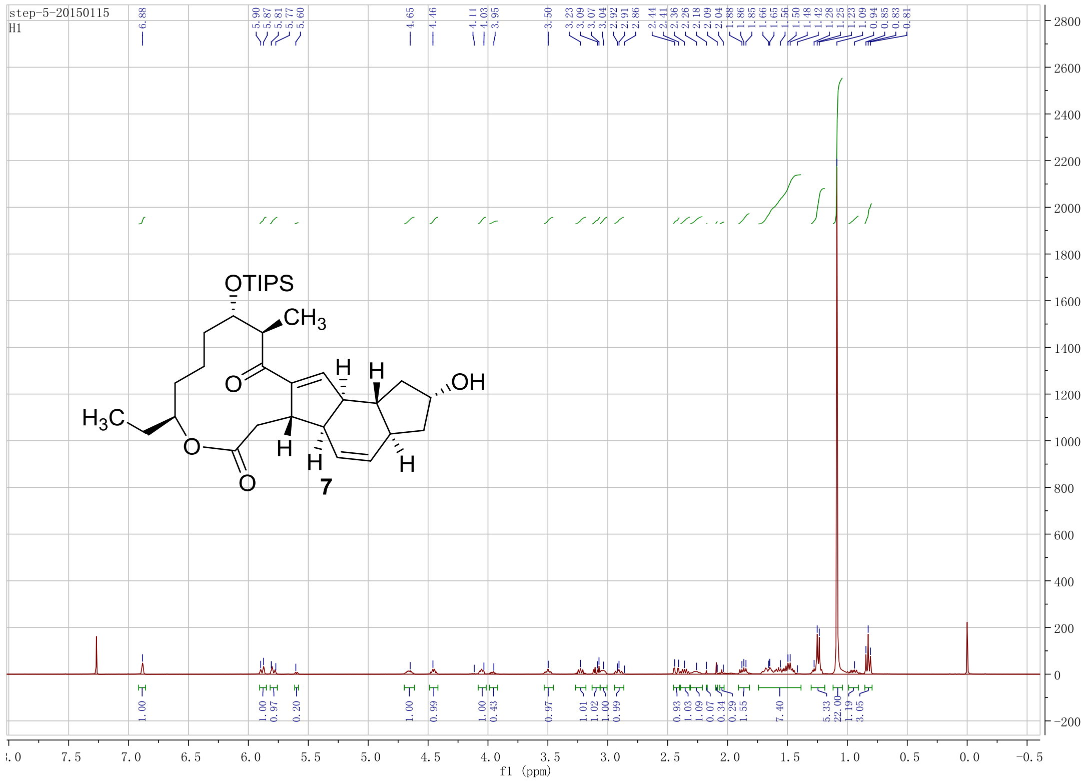

^{13}C NMR of **7**

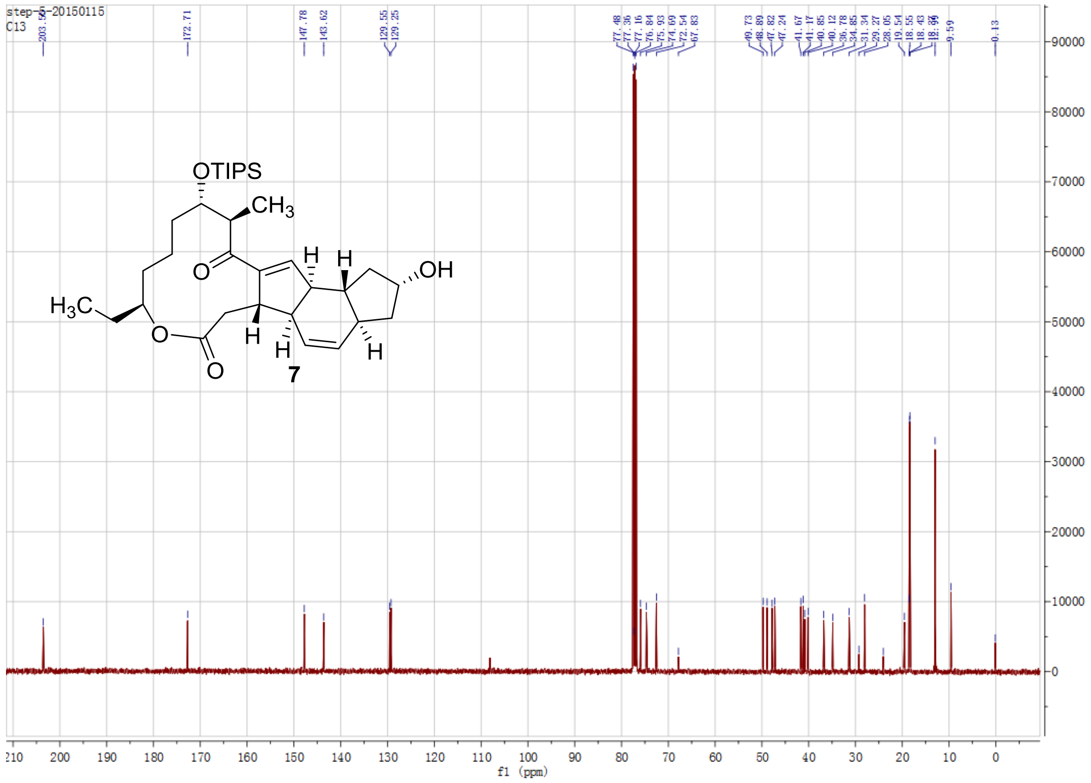

^{1}H NMR of **8**

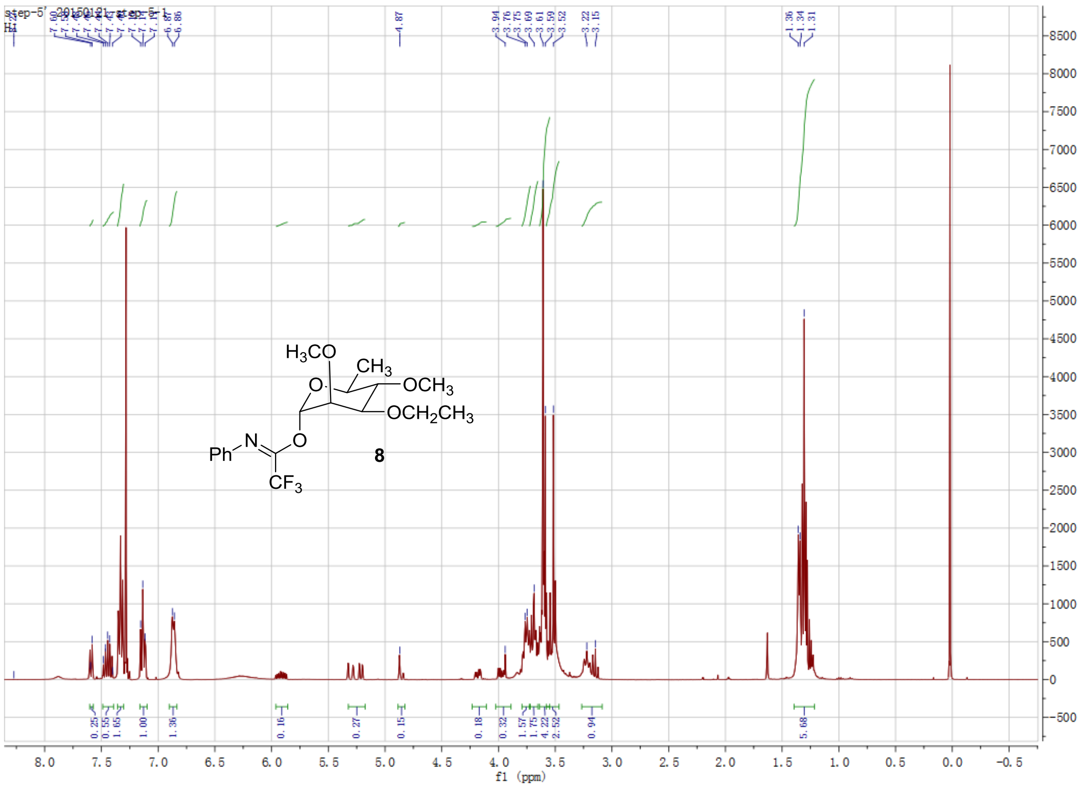

^{13}C NMR of **8**

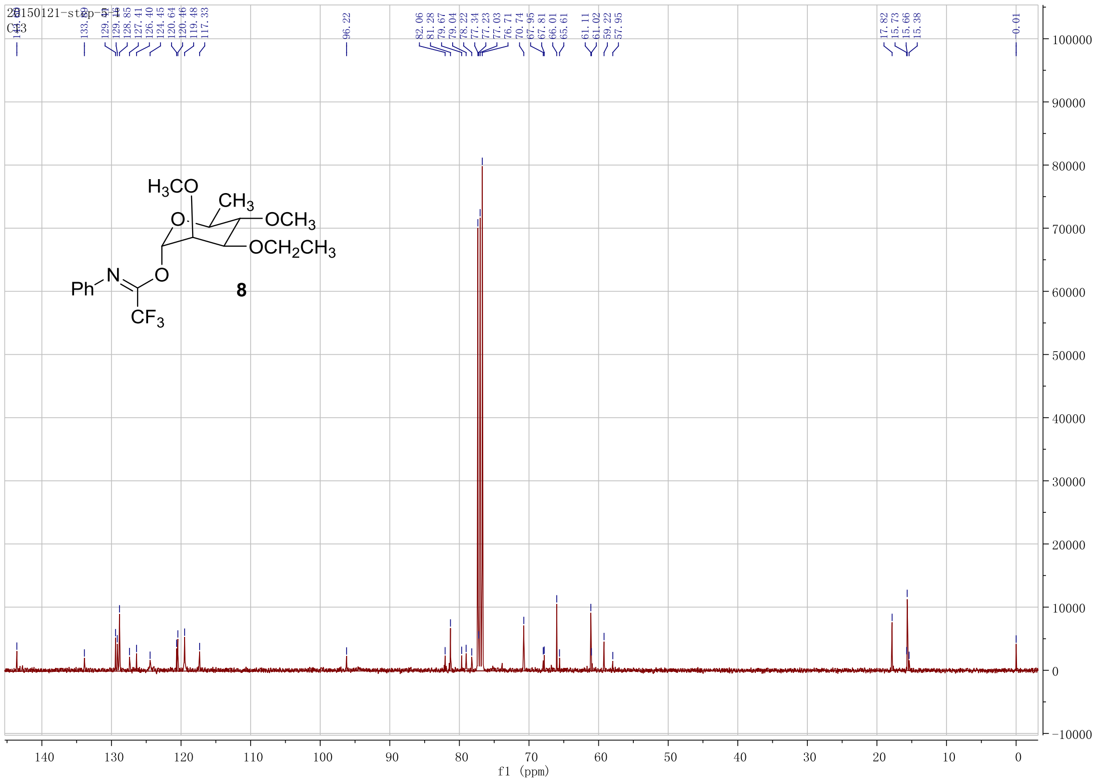

^{1}H NMR of **9**

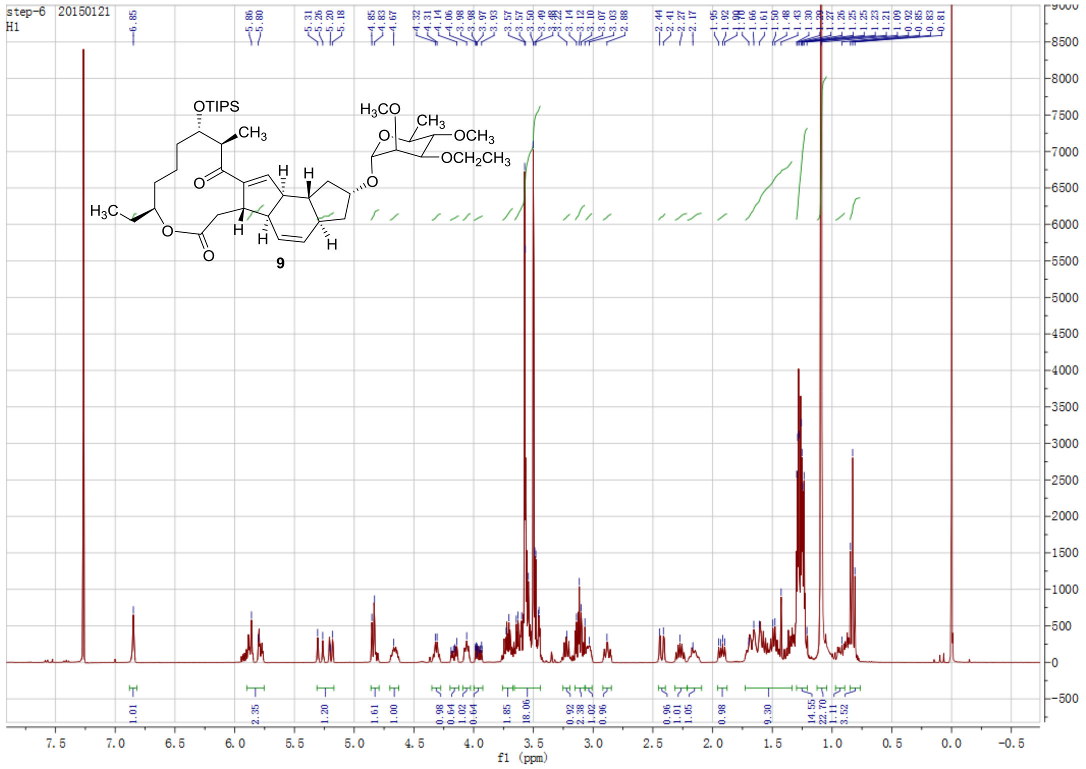

^{13}C NMR of **9**

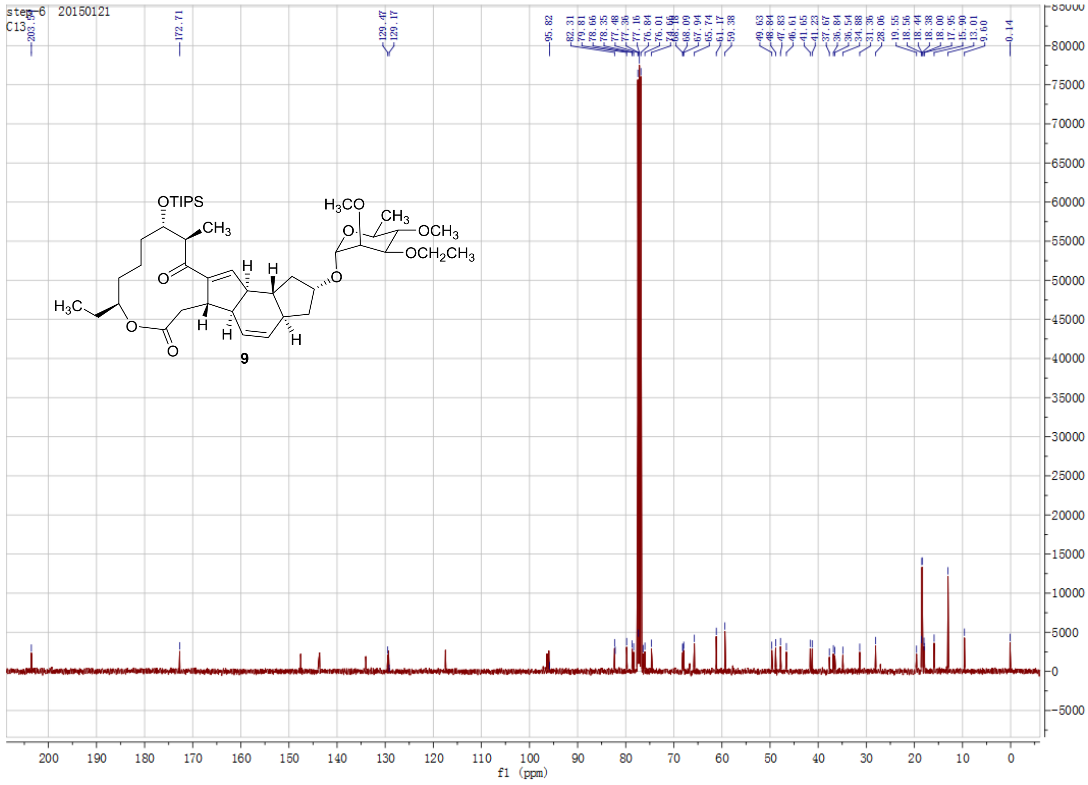

^{1}H NMR of **10**

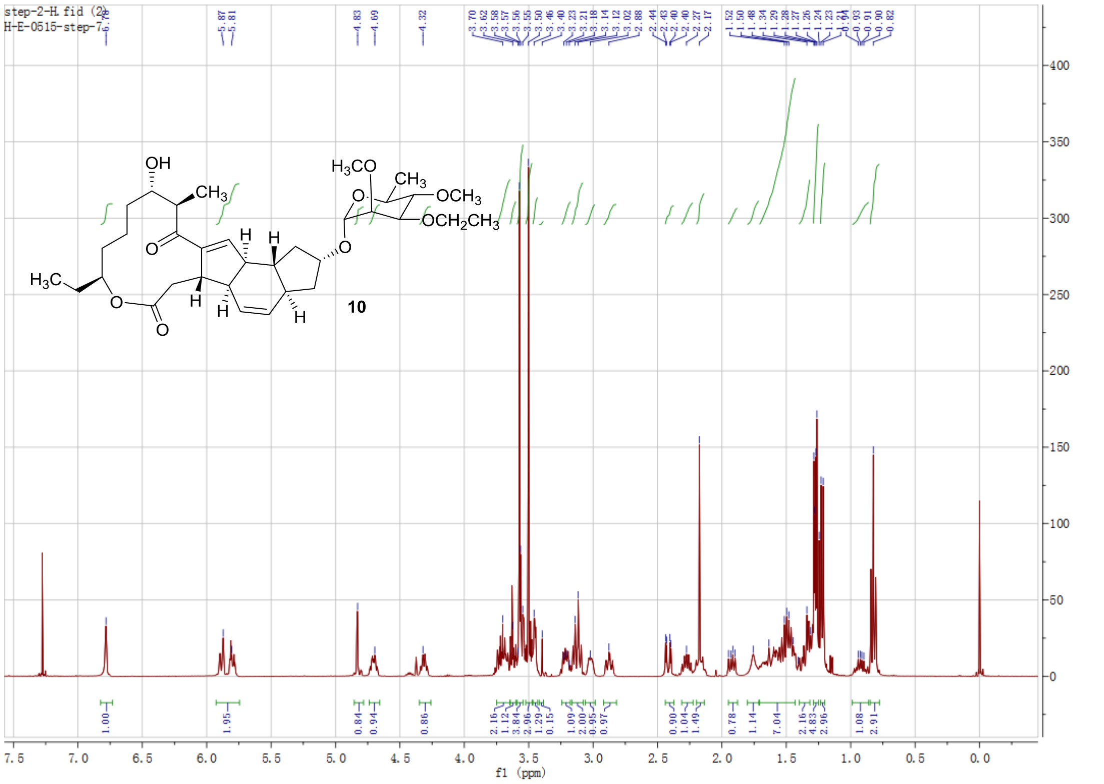

^{13}C NMR of **10**

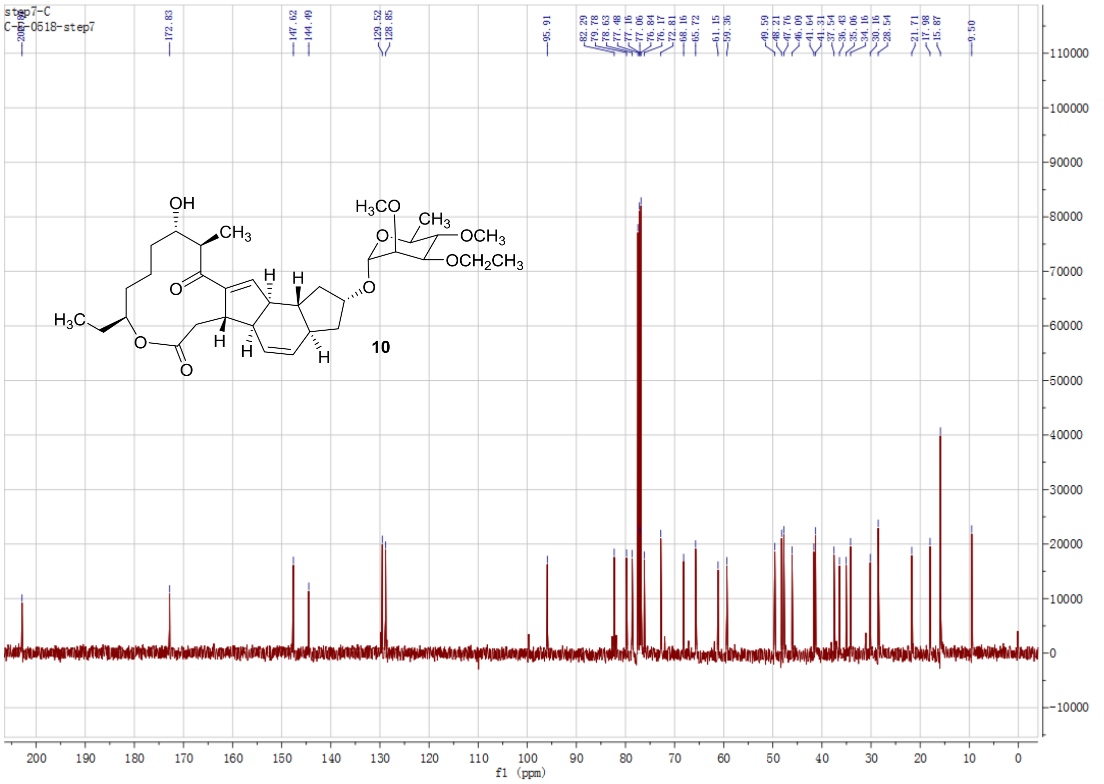

^{1}H NMR of **12**

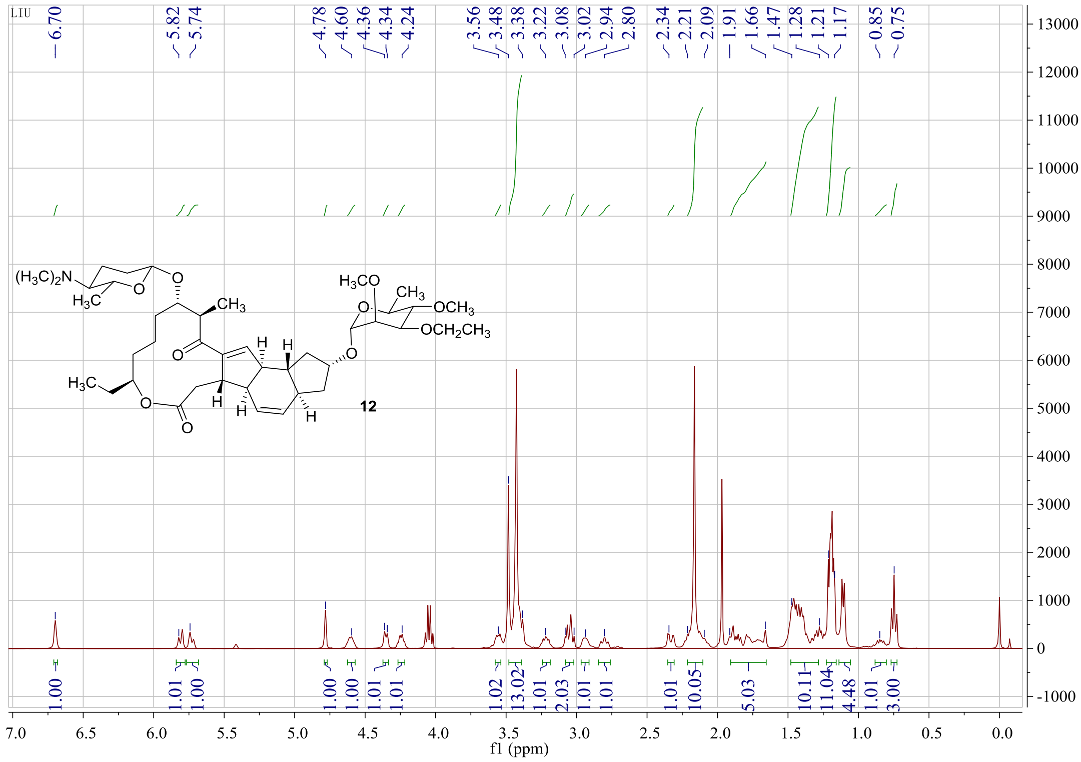

^{13}C NMR of **12**

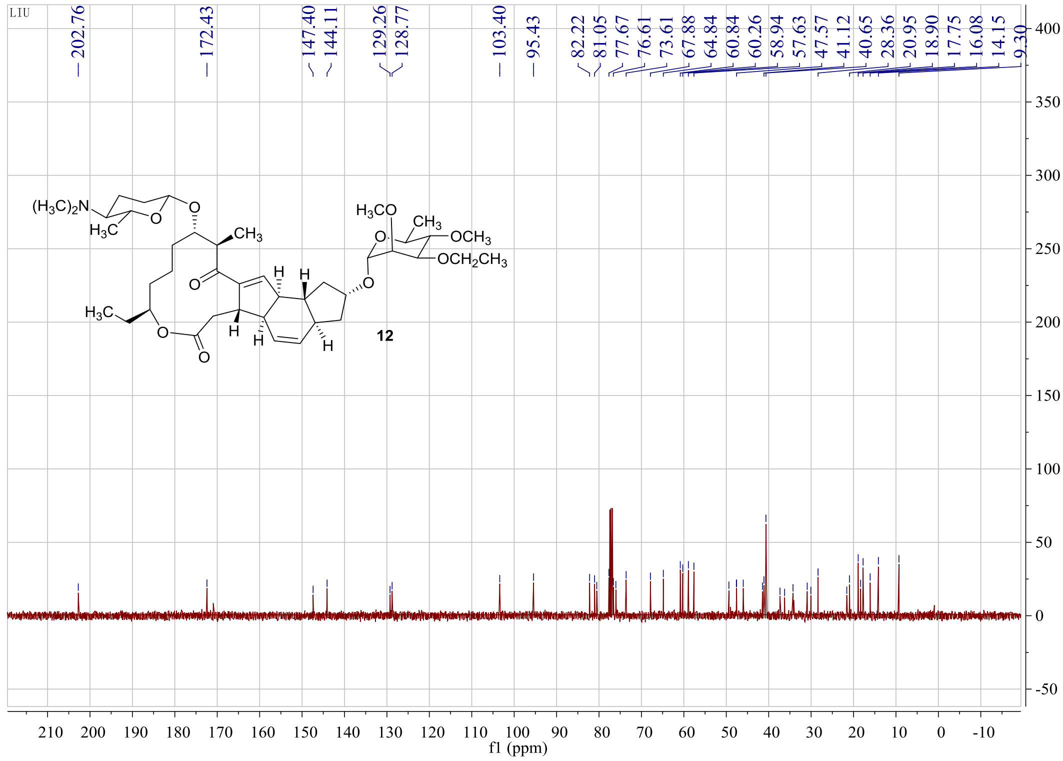

1H NMR of 3'-*O*-ethyl-5, 6-dihydro spinosyn J

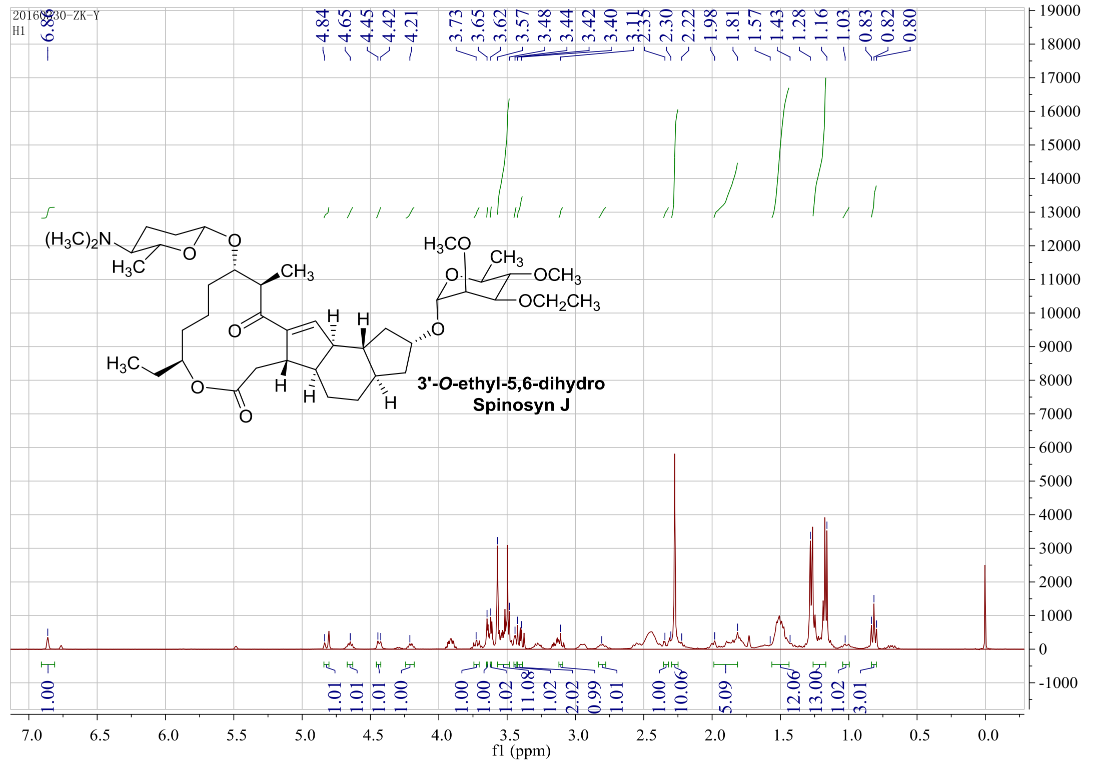

13C NMR of 3'-*O*-ethyl-5, 6-dihydro spinosyn J

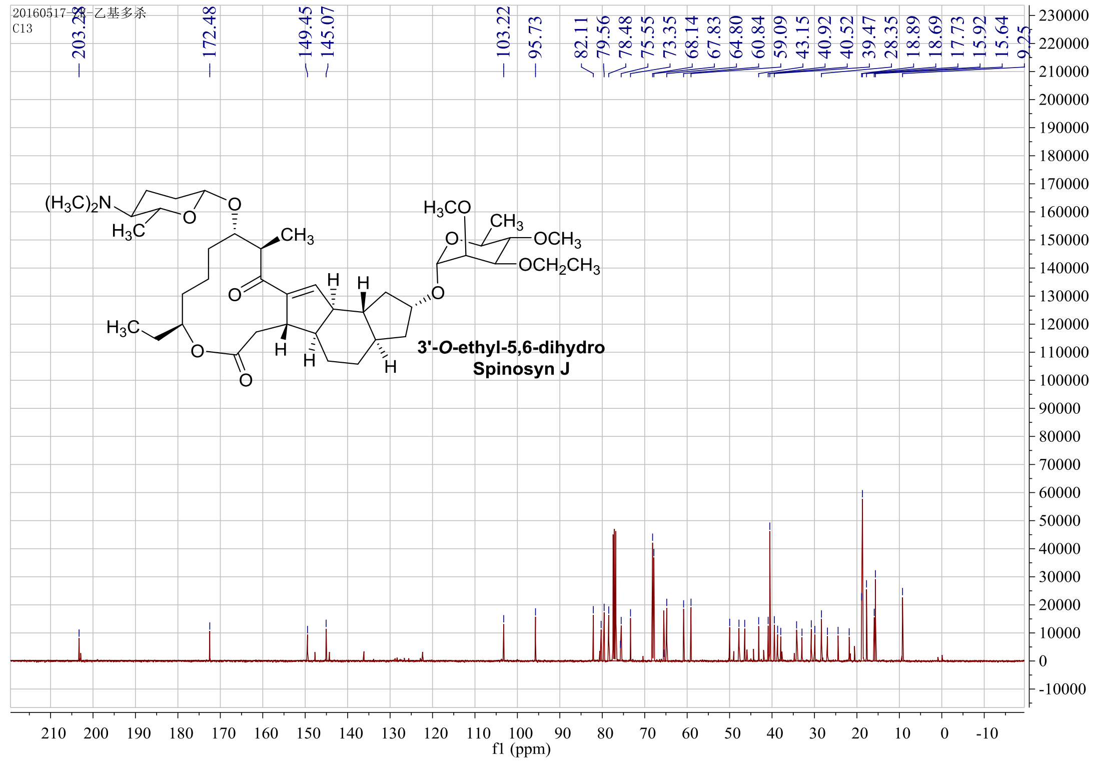

MS of **1**

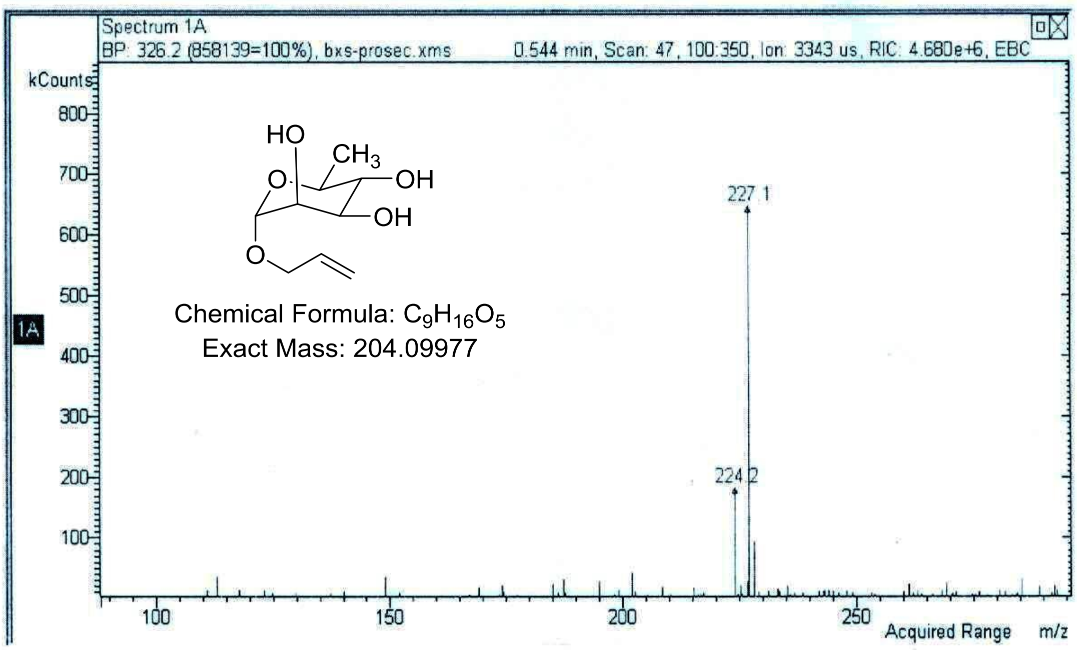

Spectrum 1A
BP: 326.2 (858139=100%), bxs-prosec.xms
0.544 min, Scan: 47, 100:350, Ion: 3343 us, RIC: 4.680e+6, EBC
kCounts
1A
227.1
224.2
Chemical Formula: C9H16O5
Exact Mass: 204.09977
Acquired Range
m/z

MS of **2**

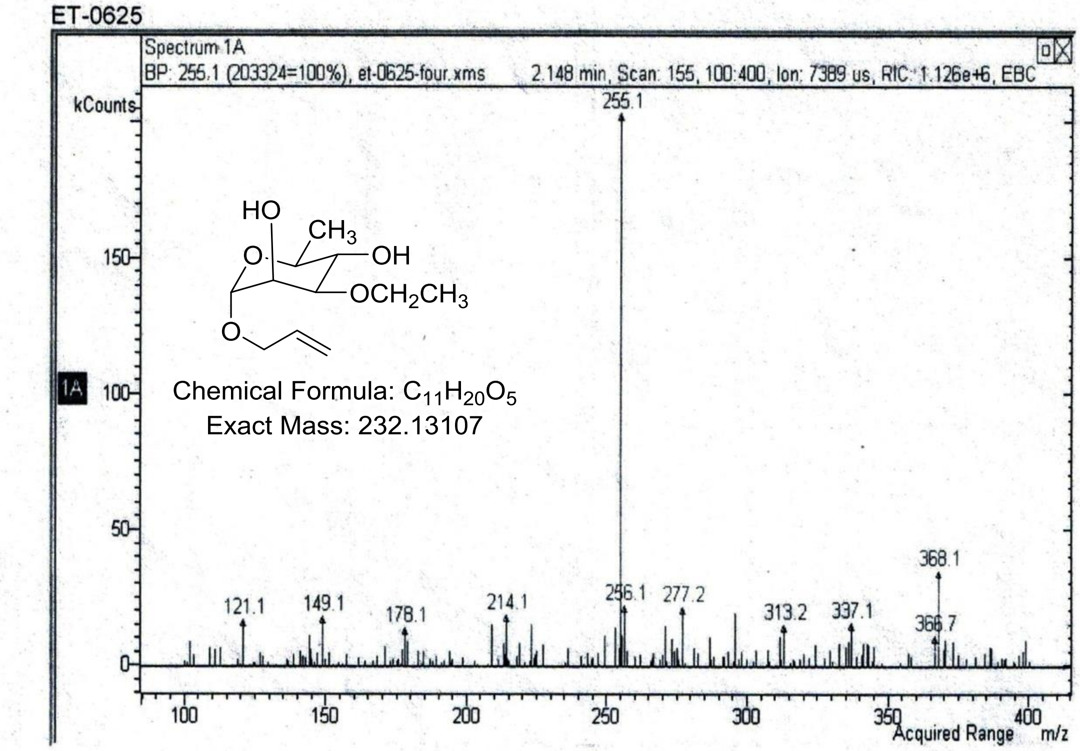

ET-0625
Spectrum 1A
BP: 255.1 (203324=100%), et-0625-four.xms
2.148 min, Scan: 155, 100:400, Ion: 7389 us, RIC: 1.126e+6, EBC
kCounts
1A
255.1
121.1
149.1
178.1
214.1
256.1
277.2
313.2
337.1
368.1
366.7
Chemical Formula: C11H20O5
Exact Mass: 232.13107
Acquired Range
m/z

MS of **3**

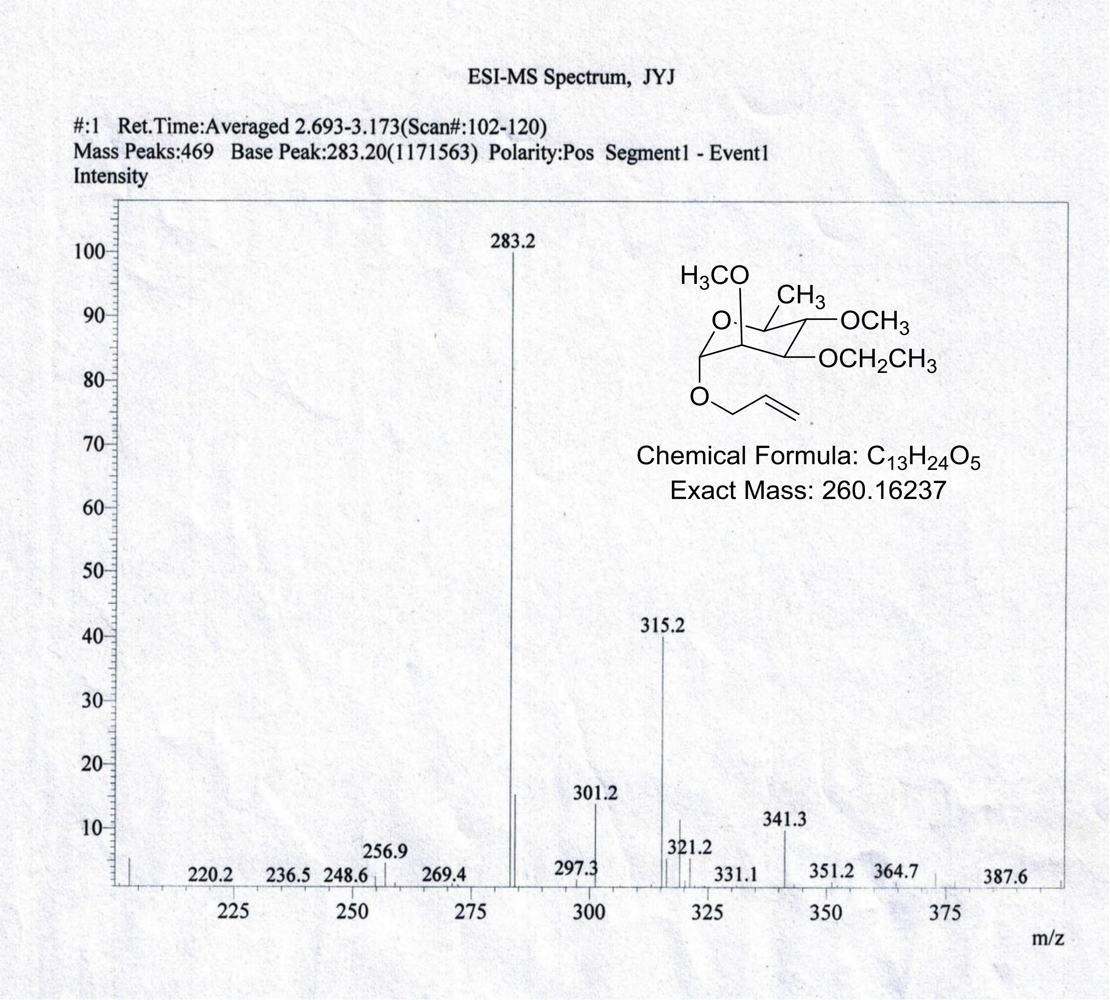
ESI-MS Spectrum, JYJ
#:1 Ret.Time:Averaged 2.693-3.173(Scan#:102-120)
Mass Peaks:469 Base Peak:283.20(1171563) Polarity:Pos Segment1 - Event1
Intensity
283.2
315.2
301.2
341.3
321.2
256.9
220.2
236.5
248.6
269.4
297.3
331.1
351.2
364.7
387.6
m/z
H3CO
CH3
OCH3
OCH2CH3
O
Chemical Formula: C13H24O5
Exact Mass: 260.16237

MS of **4**

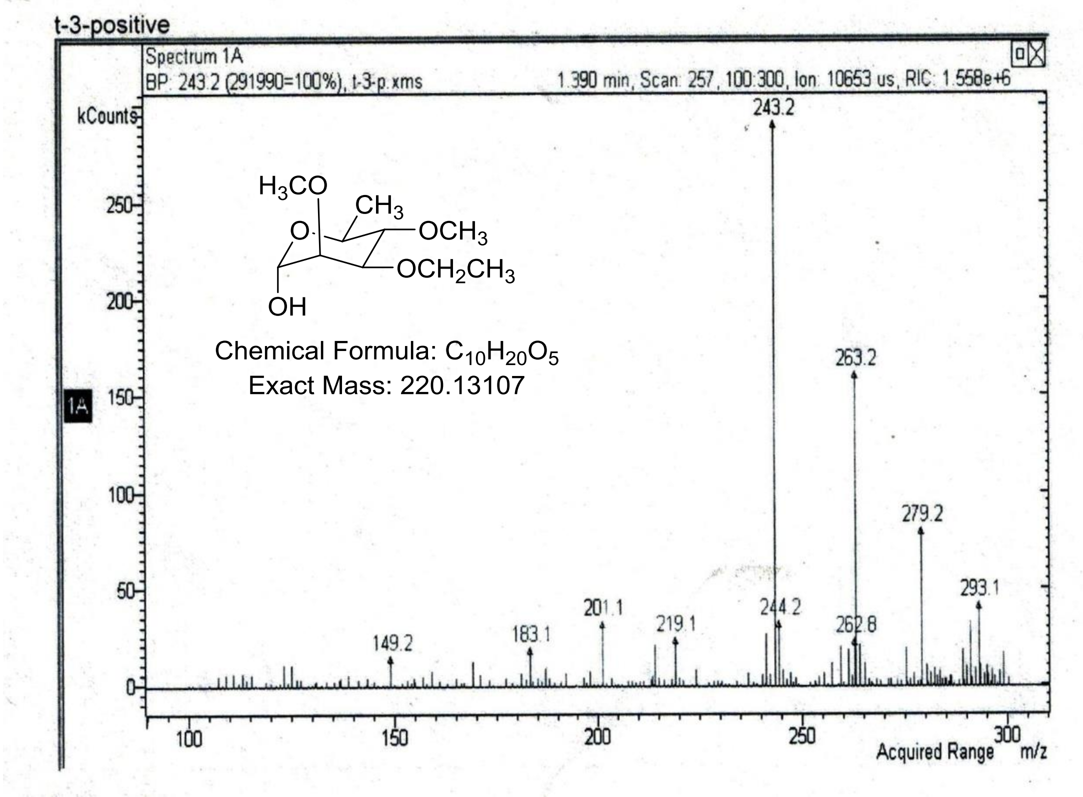

MS of **5**

Peking University Mass Spectrometry Sample Analysis Report

Analysis Info

Analysis Name	15050836_20150521_000008.d	Acquisition Date	5/21/2015 11:11:22 AM
Sample	step-3	Instrument	Bruker Apex IV FTMS
Comment	ESI Positive	Operator	Peking University

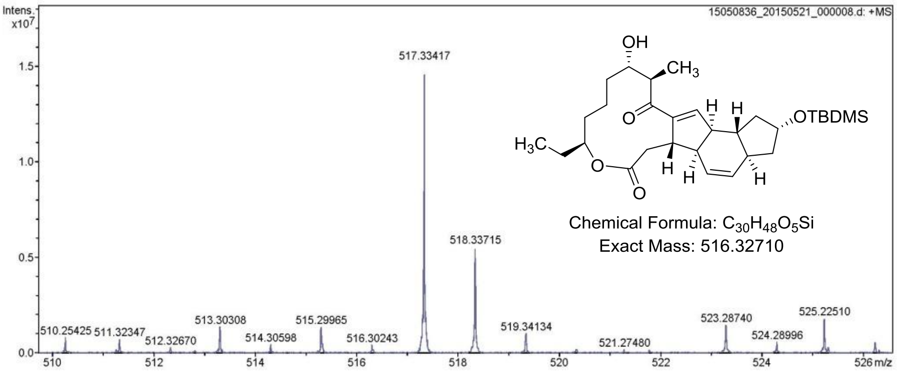

Meas. m/z	I	Formula	Score	m/z	err [mDa]	err [ppm]	mSigma	rdb	e⁻ Conf	N-Rule
517.33417	15213648	C 30 H 49 O 5 Si	100.00	517.33438	0.2	0.4	24.2	7.5	even	ok

Bruker Compass DataAnalysis 4.0 printed: 5/21/2015 11:12:28 AM Page 1 of 1

MS of **6**

Peking University Mass Spectrometry Sample Analysis Report

Analysis Info

Analysis Name	14120381_20141210_000005.d	Acquisition Date	12/10/2014 3:24:10 PM
Sample	TBS-TIPS	Instrument	Bruker Apex IV FTMS
Comment	ESI Positive	Operator	Peking University

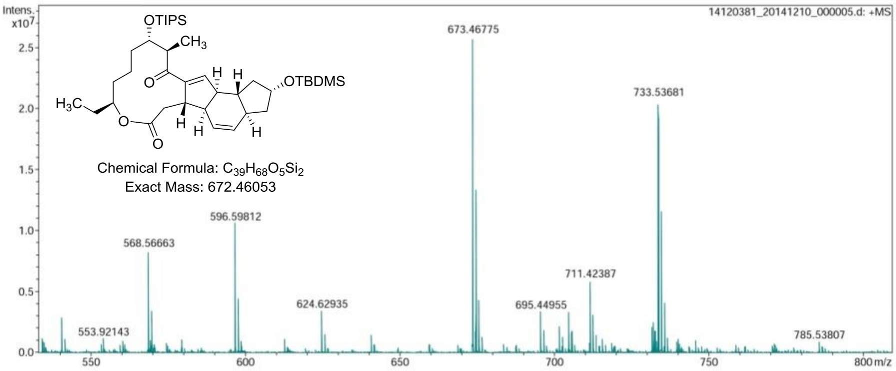

Meas. m/z	I	Formula	Score	m/z	err [mDa]	err [ppm]	mSigma	rdb	e⁻ Conf	N-Rule
673.46775	25996056	C 39 H 69 O 5 Si 2	100.00	673.46780	0.1	0.1	20.0	7.5	even	ok

MS of **7**

Peking University Mass Spectrometry Sample Analysis Report

Analysis Info

Analysis Name	15010444_20150113_000001.d	Acquisition Date	1/13/2015 4:18:19 PM
Sample	step-8-1	Instrument	Bruker Apex IV FTMS
Comment	ESI Positive	Operator	Peking University

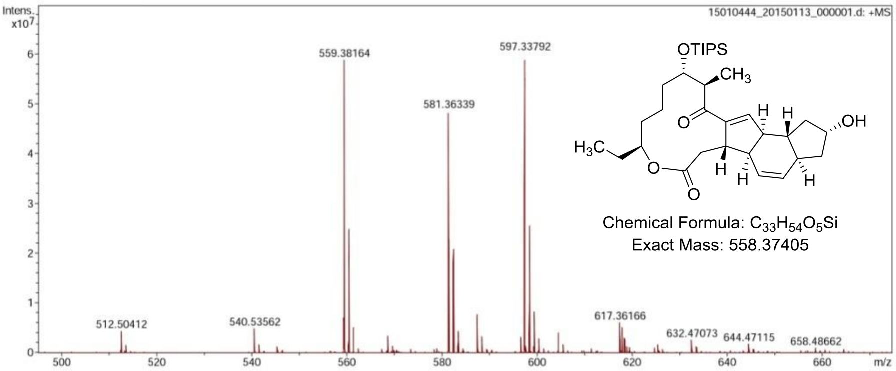

Meas. m/z	I	Formula	Score	m/z	err [mDa]	err [ppm]	mSigma	rdb	e⁻ Conf	N-Rule
559.38164	58853536	C 33 H 55 O 5 Si	100.00	559.38133	-0.3	-0.6	21.0	7.5	even	ok

MS of **8**

Peking University Mass Spectrometry Sample Analysis Report

Analysis Info

Analysis Name	15010681_20150120_000001.d	Acquisition Date	1/20/2015 8:30:29 AM
Sample	step-5-1	Instrument	Bruker Apex IV FTMS
Comment	ESI Positive	Operator	Peking University

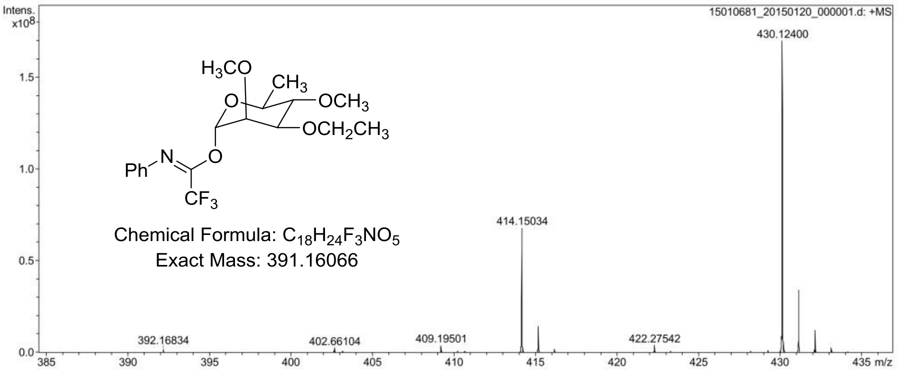

Meas. m/z	I	Formula	Score	m/z	err [mDa]	err [ppm]	mSigma	rdb	e⁻ Conf	N-Rule
392.16834	1460495	C 18 H 25 F 3 N O 5	100.00	392.16793	-0.4	-1.0	21.6	5.5	even	ok
414.15034	67993544	C 18 H 24 F 3 N Na O 5	100.00	414.14988	-0.5	-1.1	8.0	5.5	even	ok
430.12400	169979024	C 18 H 24 F 3 K N O 5	100.00	430.12382	-0.2	-0.4	14.6	5.5	even	ok

Bruker Compass DataAnalysis 4.0 printed: 1/20/2015 8:36:15 AM Page 1 of 1

MS of **9**

Peking University Mass Spectrometry Sample Analysis Report

Analysis Info

Analysis Name	15010682_20150120_000001.d	Acquisition Date	1/20/2015 8:40:19 AM
Sample	step-9-1	Instrument	Bruker Apex IV FTMS
Comment	ESI Positive	Operator	Peking University

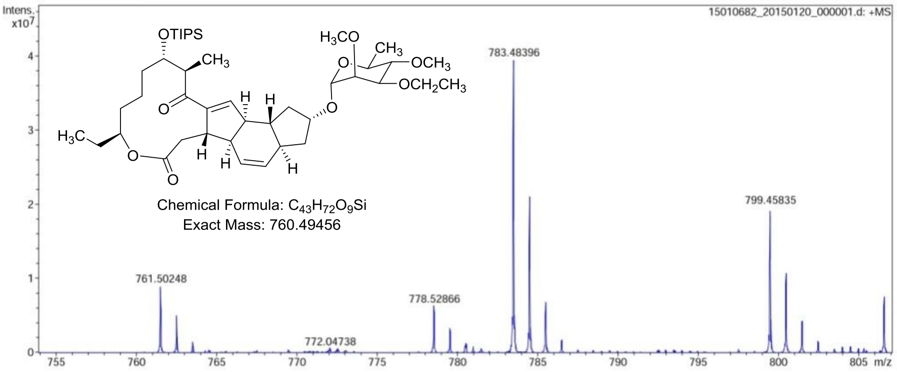

Meas. m/z	I	Formula	Score	m/z	err [mDa]	err [ppm]	mSigma	rdb	e⁻ Conf	N-Rule
761.50248	9106137	C 43 H 73 O 9 Si	100.00	761.50184	-0.6	-0.8	13.9	8.5	even	ok
783.48396	39641096	C 43 H 72 Na O 9 Si	100.00	783.48378	-0.2	-0.2	9.5	8.5	even	ok
799.45835	19726694	C 43 H 72 K O 9 Si	100.00	799.45772	-0.6	-0.8	18.7	8.5	even	ok

Bruker Compass DataAnalysis 4.0 printed: 1/20/2015 8:43:33 AM Page 1 of 1

Peking University Mass Spectrometry Sample Analysis Report

Analysis Info

Analysis Name	15010998_20150126_000001.d	Acquisition Date	1/26/2015 5:03:18 PM
Sample	step-10-1	Instrument	Bruker Apex IV FTMS
Comment	ESI Positive	Operator	Peking University

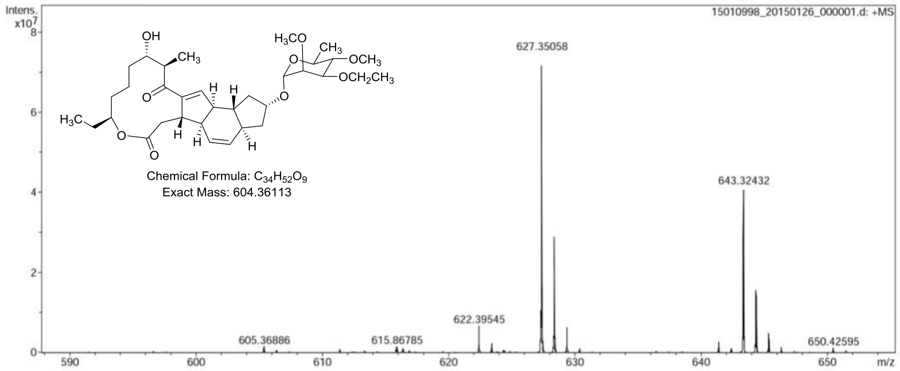

Meas. m/z	I	Formula	Score	m/z	err [mDa]	err [ppm]	mSigma	rdb	e⁻ Conf	N-Rule
605.36886	1525936	C 34 H 53 O 9	100.00	605.36841	-0.4	-0.7	12.0	8.5	even	ok
627.35058	74850640	C 34 H 52 Na O 9	100.00	627.35035	-0.2	-0.4	6.9	8.5	even	ok

Bruker Compass DataAnalysis 4.0 printed: 1/26/2015 5:04:43 PM Page 1 of 1

MS of **12**

MALDI(p),745,20150818

Analysis Info

Analysis Name	D:\Data\MALDI\2015\0818\745_0_F5_000001.d	Acquisition Date	8/18/2015 3:30:31 PM
Method	MALDI_N_100-900	Operator	
Sample Name		Instrument	solariX
Comment			

Acquisition Parameter

Acquisition Mode	Single MS	Acquired Scans	5	Calibration Date	Wed Aug 12 10:32:19
Polarity	Positive	No. of Cell Fills	1	Data Acquisition Size	2048576
Broadband Low Mass	202.1 m/z	No. of Laser Shots	10	Data Processing Size	2097152
Broadband High Mass	1400.0 m/z	Laser Power	29.6 lp	Apodization	Sine-Bell Multiplication
Source Accumulation	0.001 sec	Laser Shot Frequency	0.020 sec		
Ion Accumulation Time	0.300 sec				

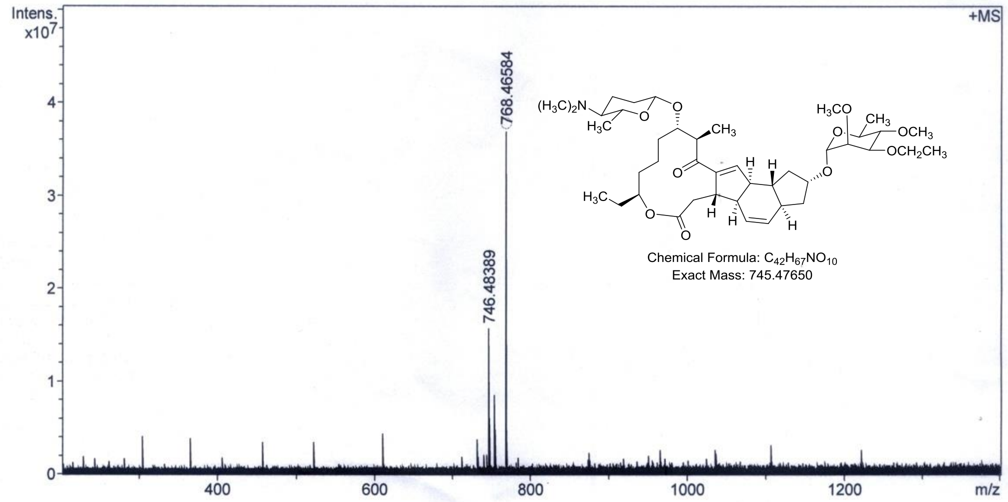

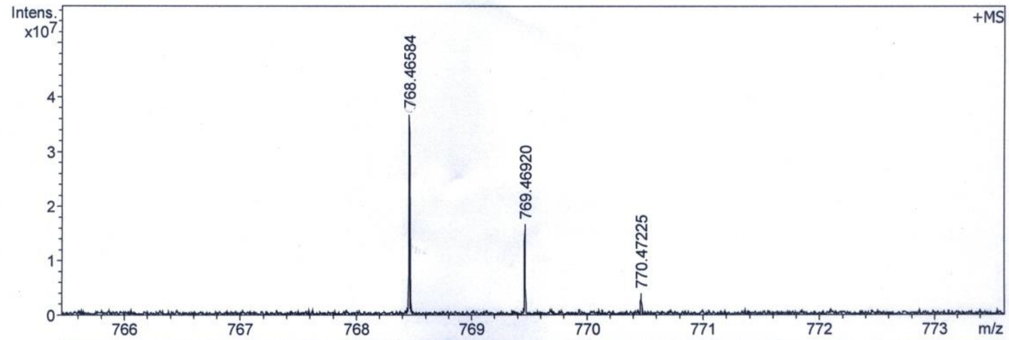

Meas. m/z	#	Ion Formula	Score	m/z	err [ppm]	Mean err [ppm]	mSigma	rdb	e⁻ Conf	N-Rule
768.465844	1	C42H67NNaO10	100.00	768.465718	0.2	-0.2	16.6	9.5	even	ok

MS of **3'-*O*-ethyl-5, 6-dihydro spinosyn J**

MALDI,ZK-Y,20160127

Analysis Info

Analysis Name	D:\Data\MALDI\2016\0127\ZK-Y_0_G14_000001.d	Acquisition Date	1/27/2016 5:01:16 PM
Method	MALDI_P_100-3000	Operator	
Sample Name		Instrument	solariX
Comment			

Acquisition Parameter

Acquisition Mode	Single MS	Acquired Scans	5	Calibration Date	Wed Jan 27 05:00:47
Polarity	Positive	No. of Cell Fills	1	Data Acquisition Size	2046576
Broadband Low Mass	202.1 m/z	No. of Laser Shots	10	Data Processing Size	2097152
Broadband High Mass	1400.0 m/z	Laser Power	26.6 lp	Apodization	Sine-Bell Multiplication
Source Accumulation	0.001 sec	Laser Shot Frequency	0.020 sec		
Ion Accumulation Time	0.300 sec				

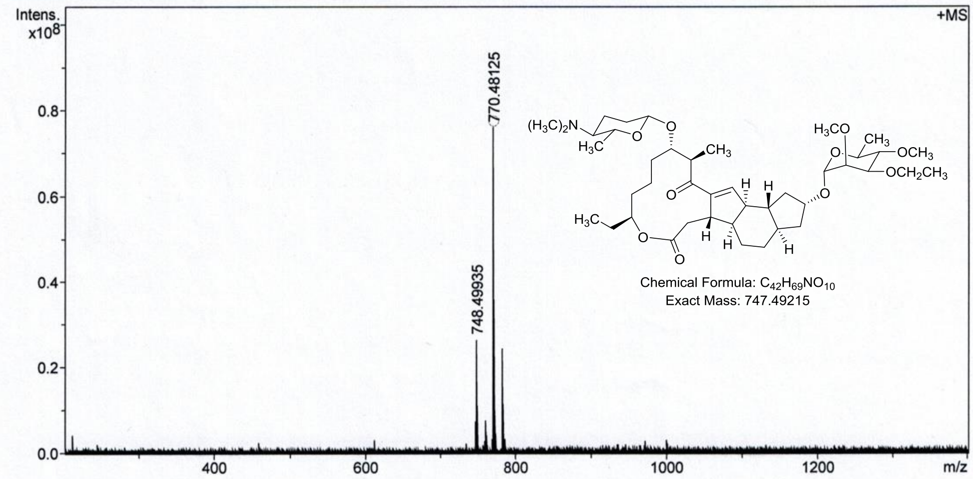

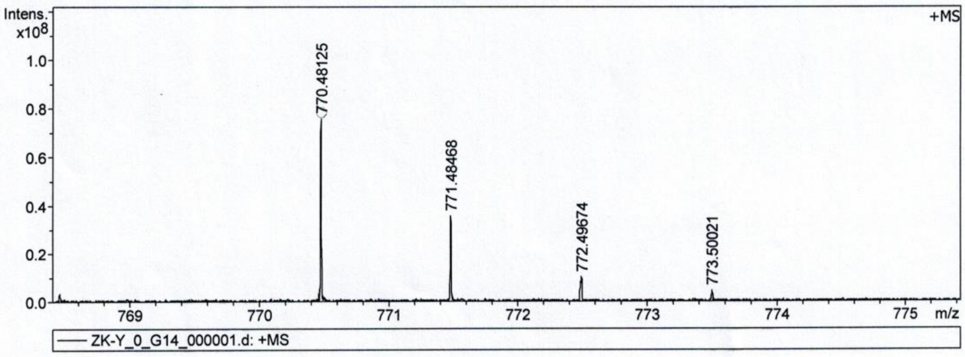

Meas. m/z	#	Ion Formula	Score	m/z	err [ppm]	Mean err [ppm]	mSigma	rdb	e⁻ Conf	N-Rule
770.481246	1	C42H69NNaO10	100.00	770.481368	-0.2	0.1	58.6	8.5	even	ok
